# Scalable Preparation of the Masked Acyl Cyanide TBS-MAC

**DOI:** 10.3390/molecules28135087

**Published:** 2023-06-29

**Authors:** Haley Hinton, Jack Patterson, Jared Hume, Krunal Patel, Julie Pigza

**Affiliations:** Chemistry and Biochemistry, University of Southern Mississippi, Hattiesburg, MS 39406, USA

**Keywords:** masked acyl cyanide, MAC, umpolung, synthon, synthetic equivalent, scalable

## Abstract

This paper describes the three-step synthesis of TBS-MAC, a masked acyl cyanide (MAC) and a versatile one-carbon oxidation state three synthon. We have developed a scalable and detailed synthesis that involves: (1) acetylation of malononitrile to form the sodium enolate, (2) protonation of the enolate to form acetylmalononitrile, and (3) epoxidation of the enol, rearrangement to an unstable alcohol, and TBS-protection to form the title compound. Both the sodium enolate and acetylmalononitrile are bench-stable precursors to the intermediate hydroxymalononitrile, which can be converted to other MAC reagents beyond TBS by varying the protecting group (Ac, MOM, EE, etc.).

## 1. Introduction

**Scheme 1:**
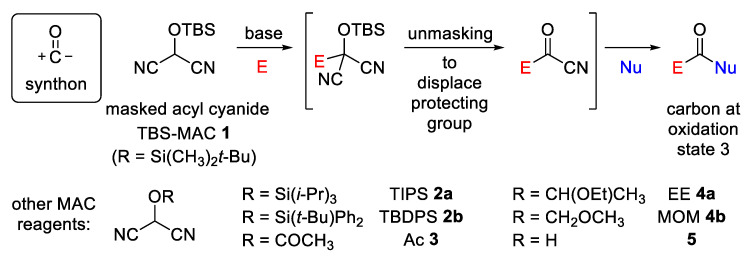
Utility of TBS-MAC **1** and other masked acyl cyanides.

The title reagent, 2-((*tert*-butyldimethylsilyl)oxy)malononitrile, also known as TBS-MAC (**1**), is an example of a masked acyl cyanide where the TBS group serves as the ‘masking’ group (Scheme 1). MAC reagents were introduced by Nemoto in the early 1990s and are represented as H-MAC-R, where MAC stands for “−C(CN)_2_O−”, H is the methine hydrogen, and R is the protecting group [1]. Protecting groups include other silyl derivatives (R = TIPS **2a**, TBDPS **2b**) [2], esters (such as R = Ac **3**), acetals (R = EE **4a**, MOM **4b**), and methoxymethyl (R = MOM **4**, Scheme 1) [3]. The unprotected and simplest masked acyl cyanide, H-MAC-H (R = H **5**, referred to as hydroxymalononitrile), is not stable alone and has not been purified [3]. Instead, it serves as an intermediate toward the formation of protected MAC derivatives. The utility of MAC reagents such as **1**–**4** is demonstrated through their umpolung reactivity [4] as both a carbonyl anion equivalent and active ester, providing value beyond the acyl anion equivalent [5]. This involves a two-step sequence of (1) deprotonation of MAC and addition of the appropriate electrophile (E), followed by (2) unmasking via removal of the protecting group to generate an acyl cyanide, which can be intercepted by a nucleophile (Nu, Scheme 1). The unmasking can also be performed in one pot, depending on the protecting group and reaction conditions. Alternatively, the protecting group can be transferred to the electrophile during the first step via migration.

The p*K_a_* of the methine hydrogen has not been reported but is likely lower than 10 as it can be deprotonated with a tertiary amine or bicarbonate base. Therefore, MAC reagents can be exploited in a variety of bond transformations, including alkylations [1,6,7], addition to imines [8,9,10,11,12,13,14] and aldehydes [15,16,17,18,19,20], as well as conjugate addition to enones [21,22] and quinone methides [23]. It has also been used as a linchpin or as a one-carbon homolog in the total synthesis of complex natural products [24,25,26,27]. Only recently has the organocatalyzed MAC addition to electrophiles been realized in an enantioselective fashion with both organocatalysts [13,14,22] and organometallic catalysts [7]. This selection of reactions involving MAC reagents highlights the utility of these one-carbon synthons. In general, the mild reaction conditions when using MAC reagents prevent unwanted side reactions such as epimerization of an α-stereocenter or β-elimination [3].

**Scheme 2:**
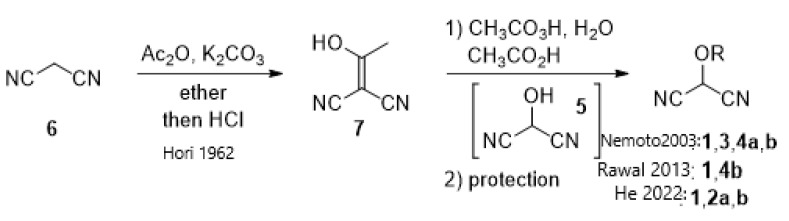
Prior syntheses of protected MAC derivatives [2,22,28,29].

TBS-MAC (**1**) was originally accessed via a 5-step synthesis [1], but this had several drawbacks, including a low overall yield, the use of a costly dehydration reagent, and the inability to prepare acylated MAC derivatives [3]. As a result, a shorter preparation of **1** (resulting in 0.3 g) was developed by Nemoto in 2003, starting from acetylmalononitrile (**7**, Scheme 2) [28]. The reaction proceeds through epoxidation of **7** and rearrangement to form unstable alcohol **5**, which can then be carried through various protection strategies. In turn, **7** was accessible from malononitrile **6** via deprotonation and acetylation, as described in an earlier report [29]. An alternative for conversion of **6** to **7** using acetyl chloride and triethylamine has also been developed [30]. In 2013, Rawal presented a well-detailed conversion of **7** to **1** (resulting in 0.9 g) using a similar strategy as Nemoto [22]. In 2022, the Aitken group found higher yields for **7**→**1** using TBSOTf instead of TBSCl as the silylating reagent [2].

From our experiences in the syntheses of both **1** and **7**, we have found the following complications: (a) bases such as triethylamine and potassium carbonate were difficult to remove, or products required multiple recrystallizations to obtain pure **7**, (b) using acetyl chloride as the acetylating reagent necessitated aqueous workup and separation of **7** or its precursor metal enolate from the water was not complete, (c) reported large scale syntheses for the first step were not easily scaled down or alternatively scaled up for the second step, and (d) some procedures were not detailed enough. These factors contributed to inconsistent yields and reproducibility in our hands. Our group’s interest in TBS-MAC (**1**) is from our research in the development of new organocatalyzed reactions and the synthesis of small molecule inhibitors of the HIV integrase enzyme [20,31,32]. Due to our need for TBS-MAC on several projects, a reliable and scalable synthesis of both acetylmalononitrile (**7**) and TBS-MAC (**1**) was required to access these valuable synthons.

## 2. Results and Discussion

### 2.1. Synthetic Considerations

**Scheme 3:**
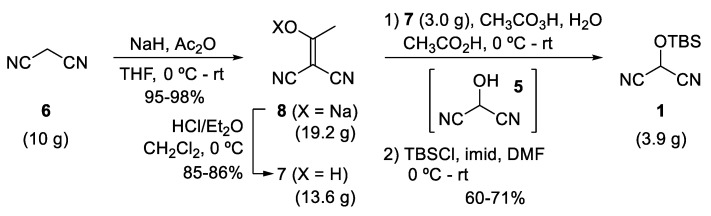
Three-step synthesis of TBS-MAC **1** from malononitrile **6**.

Our route is summarized in Scheme 3 and utilizes three total steps which are readily scalable (our maximum scales and variations in yield are noted in the scheme). Detailed reaction conditions are included in the Materials and Methods, and accompanying pictures of reactions/techniques and spectra are included in the Supplementary Material. Step 1 is the conversion of malononitrile **6** to sodium enolate **8**. This reaction is performed on either 5 g or 10 g scales, resulting in 9.6 g or 19.2 g of **8**, respectively. Step 2 involves the protonation of enolate **8** to form acetylmalononitrile **7**. Both steps take advantage of solubility differences to avoid aqueous workups and require no purification. Both **7** and **7** are bench-stable solids as well.

For the third step, we followed a similar procedure to those described prior [2,22,28] but found we could reduce the volume of unstable peroxide by half. We also determined that it can be broken up over two days, with storage of **5** in the aqueous acidic solution overnight in the freezer, providing an alternative for breaking up a long reaction.

### 2.2. Step 1—Sodium Enolate 8 (Sodium 1,1-dicyanoprop-1-en-2-olate)

**Figure 1:**
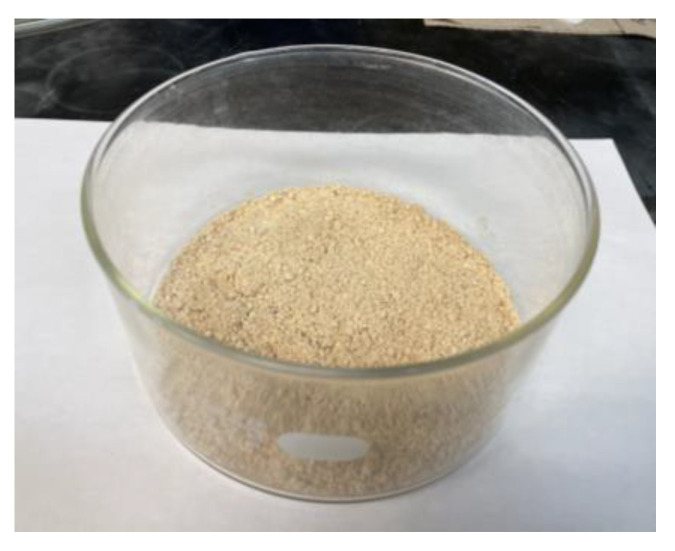
Sodium enolate **8**.

The conversion of malononitrile **6** to acetylmalononitrile **7** occurs through a metal enolate, the identity of which depends on the base used. This enolate was not isolated in prior syntheses [2,22,28,29,30]. As part of avoiding water solubility issues of products, we chose to use sodium hydride (NaH) as the base and to isolate the enolate. Deprotonation of malononitrile **6** with NaH in THF was completed at 0 °C, followed by the addition of acetic anhydride at the same temperature (Table 1). Excess base (2.0 equiv) was used since two deprotonations are required, **6**→**9** and the acetylated intermediate (not shown) to form enolate **8**. We found the time to be scalable, such that at double the scale, the time was doubled in both steps (for example, entry 1 to entry 2, Table 1). Despite the thick, crude reaction, volatiles could be removed by rotary evaporation without bumping. This yielded only two products—the desired sodium enolate **8** and sodium acetate, which were separated by a slurry with acetone that solubilizes enolate **8** but leaves behind the NaOAc byproduct. Simple suction filtration and removal of volatiles resulted in sodium enolate **8** as a tan solid (Figure 1).

All entries in Table 1 used NaH that had been washed with hexanes prior to use, as we noticed that the consistency of the final product would vary otherwise. We also found the volume and number of acetone washes were important. Two acetone slurries were sufficient on a smaller scale (entry 1) but not on a larger scale (entry 2). Using two larger slurries (entry 2) was more effective than three smaller ones (entry 3). The mixture is very thick, which explains why sufficient acetone is required and why more slurries were required on a larger scale (entry 4). A fourth wash gave a minimal increase in yield (95% in entry 4 vs. 98% in entry 5). Enolate **8** could be stored in a closed container on the lab bench for at least a year without showing any degradation in color or purity.

### 2.3. Step 2—Acetylmalononitrile 6 (2-(1-Hydroxyethylidene)malononitrile)

**Figure 2:**
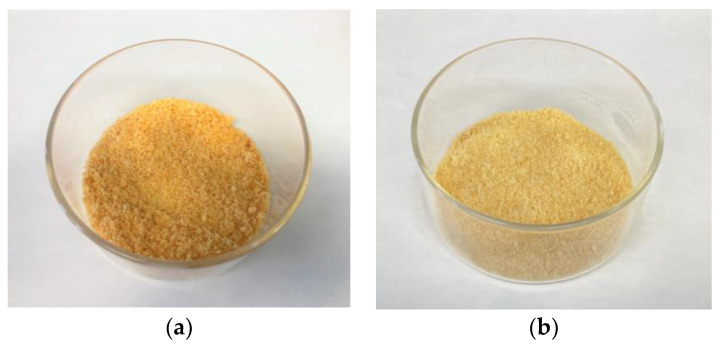
Acetylmalononitrile **7** before (**a**) and after (**b**) a hexanes slurry.

The conversion of sodium enolate **8** to acetylmalononitrile **7** involves protonation with hydrochloric acid (Table 2). By using an organic solvent rather than an aqueous solution of HCl, we avoid the water solubility of **7**. The stirring time after complete addition can be kept to a minimum at each scale (entries 1 and 2, Table 1), resulting in only acetylmalononitrile **7** and NaCl, the latter of which is filtered. We utilized the slurry procedure as in the prior step, except with DCM. Unlike the prior step though, additional DCM slurries did not provide any appreciable material. We have found that while the ^1^H and ^13^C NMR of solid **7** match the expected product, the consistency of the solid is somewhat sticky (Figure 2a). A simple hexanes slurry provided acetylmalononitrile **7** as a tan/yellow-orange solid (Figure 2b) in good yield on both scales (entries 1 and 2, Table 2).

### 2.4. Step 3—TBS-MAC 1 (2-((tert-Butyldimethylsilyl)oxy)malononitrile)

The third step required the most optimization. Following the general procedure developed by Nemoto [28] and provided in more detail by Rawal [22] and Aitken [2], we undertook the oxidation of acetylmalononitrile **7** by peracetic acid (CH_3_CO_3_H, Table 3). The mechanism involves epoxidation of **7**, followed by ring opening and rearrangement to form the unstable hydroxymalononitrile **5**. On the small scales originally reported [22,28] (0.75 and 0.2 g, respectively), the acids solution was added dropwise via pipette to **7** stirring in water at room temperature. On larger scales, we noticed that this reaction heated up, which resulted in a darker yellow color. Therefore we found it best to add the acids solution dropwise via an addition funnel to **7** in water at 0 °C. We have successfully reduced the volume of peroxide by half (3.3 mL CH_3_CO_3_H solution per gram **7** compared to 6 or 7 mL) as well as the amount of water and acetic acid (17 mL per gram **7** compared to 35 or 44 mL). This ultimately facilitates safer and more rapid removal of volatiles.

The concentration of volatiles to provide intermediate **5** is performed on a rotary evaporator followed by a high vacuum with a blast shield in front of each setup due to the risk of explosion (as noted in [2,22,28] also). Removal of any remaining acids is essential as they greatly impact the yield of **1** if >5%. To ensure this, **5**, which was a whitish/pale yellow oil/solid, was checked by ^1^H and ^13^C NMR in DMSO-d_6_ (acetic acid can be observed and is tolerated below 5%). Ultimately, the care taken during the synthesis of intermediate **5** is what determines the overall success of the reaction. We have also found that **5** can be stored overnight in the freezer in the aqueous acids solution and used the following morning without much detriment in yield (only 5–7%). This helps to break up what is otherwise a long day in converting **7**→**[5]**→**1**. This inspiration was taken from the Ma group, which describes the storage of **5** this way and its use directly in further reactions in the acidic solution [33]. 

**Figure 3:**
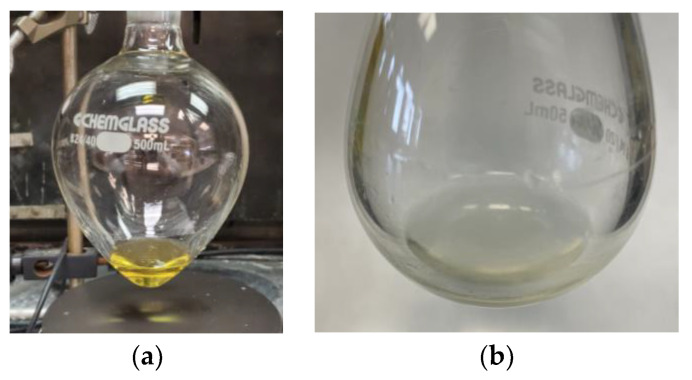
TBS-MAC **1** before (**a**) and after (**b**) chromatography.

We also undertook an examination of the protection step (second arrow in Table 3). Entry 1 represents a 0.75 g scale in which DMF is added to crude **5**, cooled to 0 °C, and then treated with TBSCl, followed immediately by imidazole in three portions (61% crude yield). We increased the reaction time (entry 2) and decreased the concentration of DMF (entry 3), with the latter having a more significant increase in yield (68% crude yield). Reducing the reaction time at 0 °C with more time at room temperature (entry 5) did not impact the yield, but the reverse order of imidazole and TBSCl was detrimental (entry 6). Therefore using the ideal conditions as in entry 3, we carried out the reactions at 2× and 4× the scale (entries 6 and 7, respectively, providing crude **1** as a yellow/orange oil, Figure 3a). Purification effectively removes the byproduct TBSOH, which is more polar and easily separated by column chromatography. TBS-MAC **1** is obtained as a colorless oil (Figure 3b) which can be stored indefinitely in the freezer and in the refrigerator while being used.

## 3. Materials and Methods

### 3.1. General Experimental

Starting materials were purchased from commercial vendors and used without purification unless noted. Unless otherwise noted, all reactions were carried out using flame-dried glassware and standard syringe, cannula, and septa techniques under an atmosphere of nitrogen (nitrogen balloon). Tetrahydrofuran (THF), diethyl ether (Et_2_O), and dichloromethane (CH_2_Cl_2_) were dried by passage through a column of activated alumina on an Innovative Technologies system. Analytical thin-layer chromatography was performed using Sorbent Technologies 250 μm glass-backed UV254 silica gel plates. The plates were first visualized by fluorescence upon 254 nm irradiation, then by an iodine chamber or one of the following stains followed by heating: phosphomolybdic acid or ceric ammonium molybdate. Flash column chromatography was performed using Sorbent Technologies silica gel (40–63 µm, pore size 60 Å) with solvent systems indicated. Buchner funnel filtrations were performed using a small Chemglass diaphragm pump (10 torr). Solvent removal was affected using a Buchi R3 rotary evaporator with a Fisher diaphragm pump (70 torr, water bath set to 30 °C unless otherwise noted). Further solvent removal under high vacuum was accomplished using a Welch direct drive vacuum pump (0.01 torr). All yields refer to isolated material that is chromatographically (TLC or HPLC) and/or spectroscopically (^1^H NMR) homogenous. All melting points were taken with a Thomas Hoover melting point apparatus and are uncorrected. Infrared spectra were recorded on a Nicolet Nexus 470 FTIR spectrometer as neat oils or solids. Proton nuclear magnetic resonance spectra (^1^H NMR) were recorded on a Bruker UltraShield Plus 400 MHz spectrometer and are recorded in parts per million (δ) from internal chloroform (7.26 ppm) or dimethylsulfoxide (2.50 ppm) and are reported as follows: chemical shift (multiplicity (br = broad, s = singlet), integration). Carbon NMR data (^13^C NMR) were recorded on a Bruker UltraShield Plus 100 MHz spectrometer and are recorded in parts per million (δ) from internal chloroform (77.0 ppm) or dimethylsulfoxide (39.5 ppm) and are reported as follows: chemical shift. Purity by ^1^H NMR was obtained by QNMR [34] using the standard 1,3,5-trimethoxybenzene purchased from Oakwood Chemicals, whose purity was noted at 100% from the certificate of analysis based on the lot number from the vendor.

### 3.2. Synthetic Procedures and Analytical Data

The reactions listed below are at the largest scales for each of the three compounds. Considerations for running at half the scale are noted after the paragraph (and discussion can also be found in the Tables in the Results and Discussion). Pictures of the key reaction steps can be found in Supplementary Material and have an ‘S’ noted in the Figure name. For example, Figure 1 corresponds to the manuscript, but Figure S1 would be in Supplementary Material. RBF = round bottom flask.

*Sodium 1,1-dicyanoprop-1-en-2-olate* (**8**). Sodium hydride (12.2 g of a 60% dispersion in oil, equal to 7.3 g, 305 mmol, 2 equiv) was added to a 500 mL, 24/40 RBF fitted with a 4.0 cm stir bar and septum, and balloon. To the NaH was added 40 mL hexanes via syringe. The flask was swirled, and the solids were allowed to settle. The hexanes were removed using a separate syringe, leaving behind the solid sodium hydride. This procedure was repeated two additional times. Then THF (180 mL of 190 mL, 0.8 M total) was cannulated into the reaction flask. The flask was topped with a reducing adaptor to a 50 mL pressure-equalizing addition funnel. The entire setup was placed in an ice-water bath for at least 15 min. To a separate 100 mL 14/20 strawberry flask with a 1.5 cm stir bar was added malononitrile **6** (10.0 g, 151 mmol, 1 equiv, used as purchased as a brown solid), followed by a septum and balloon, and THF (remaining 10 mL) via syringe. It was stirred for ~5 min until the malononitrile was dissolved and then cannulated into the addition funnel (Figure S1A). The stopcock was opened to achieve a rate of 1 drop every 5–6 s (bubbling noted in the vortex of stirring that subsides within 5 s, Figure S1B). Upon complete addition of the malononitrile, an extra 1 mL of tetrahydrofuran was used to rinse the flask, then added to the reaction at the prior rate. The tan reaction was stirred for an additional 1 h in the ice-water bath (Figure S1C). Acetic anhydride (14.4 mL, 152 mmol, 1 equiv) was added into the addition funnel via a syringe. The stopcock was opened to achieve a rate of 1 drop every 5–6 s (more vigorous bubbling was noticed that does not subside between drops, Figure S1D). After the complete addition of acetic anhydride, the reaction becomes a very thick slurry (Figure S1E). It was stirred at a higher rpm in the ice-water bath for 30 min and then at rt for 30 min. The stir bar was removed, and the reaction flask was placed on rotovap to remove volatiles (bumping was not observed despite the thick mixture, Figure S1F). The stir bar was added back to the flask, and the solid residue was stirred with acetone (200 mL) for 15 min (Figure S1G). The slurry was vacuum filtered using a diaphragm pump attached to a 105 mm Buchner funnel with 90 mm Whatman #1 filter paper on a 500 mL side arm filter flask (Figure S1H). The filtrate was set aside. The filter cake was scraped into a 600 mL beaker with the original stir bar (Figure S1I) and resubmitted to the same acetone slurry/filter procedure an additional three times (3 × 200 mL acetone, the last slurry results in 0.5 g more material and is optional). The off-white filter cake consisting of sodium acetate was discarded. The combined filtrates were transferred to a 1000 mL, 24/40 RBF, and the solvent was removed by rotary evaporation to afford sodium 1,1-dicyanoprop-1-en-2-olate (**8**) as a tan solid (19.2 g, 148 mmol, 98% yield, Figure 1). The following analytical data were obtained: mp—began to darken in color and decompose at 181.0 °C; IR (solid ATR) 2923, 2853, 2215, 2192, 2180, 1561, 1374, 1343, 1264, 967, 628, 540 cm^−1^; ^1^H NMR (400 MHz, DMSO-d_6_) δ 1.84 (s, 3H); ^13^C NMR (100 MHz, DMSO-d_6_) δ: 190.0, 123.3, 121.1, 47.0, 25.6. Sodium enolate **8** showed 92% purity by QNMR; see Supplementary Material.

For reactions on a half scale, i.e., 5.0 g malononitrile **6**, all amounts and times were halved, aside from cooling times of 15 min while in an ice-water bath. The number of acetone slurries was 2 × 100 mL.

*2-(1-Hydroxyethylidene)malononitrile* (**7**). Sodium enolate (**8**) (19.2 g, 148 mmol, 1 equiv) was added to a flame-dried 500 mL 24/40 RBF equipped with a 4.0 cm stir bar and topped with a septum and balloon. DCM (82 mL, 1.8 M) was added to the flask via cannula and stirred. An oven-dried 125 mL pressure-equalizing addition funnel was added to the top of the flask, and the septum and balloon were replaced. The reaction flask was placed in an ice-water bath for 15 min. Then 2 M HCl in Et_2_O (82 mL, 1.1 equiv) was poured into the addition funnel through a glass funnel, and the contents were added to the reaction flask at a rate of 1 drop/sec (Figure S2A). After the addition was complete, the reaction was stirred for an additional 30 min at 0 °C and was then removed from the ice bath. The stir bar was removed, and the reaction was vacuum filtered to remove the insoluble NaCl salt using the setup described for **8**. The filtrate was set aside. The filter cake was scraped into a 400 mL beaker, and the original stir bar was added, followed by DCM (100 mL). The solid was stirred for 15 min and then poured back into the Buchner funnel (Figure S2B). The filtrates were combined and transferred to a 500 mL 24/40 flask and concentrated by rotovap (Figure S2C) to provide **7** as a slightly sticky, tan/orange solid (14.9 g, 93% yield, 90% purity by QNMR, Figure 2a). The solid and original stir bar were added to a 400 mL beaker and slurried with 100 mL hexanes for 30 min. The solid was filtered using the prior Buchner funnel setup, spread out in a glass-crystallizing dish, and then dried in the hood overnight. This afforded 2-(1-hydroxyethylidene)malononitrile (**7**) as a tan/orange solid (13.6 g, 126 mmol, 85% yield, Figure 2b). The following analytical data were obtained: mp 137.2–139.4 °C; IR (solid ATR) 3047, 2611, 2240, 2224, 1600, 1400, 1360, 1333, 1302, 1228, 971, 804, 627 cm^−1^; ^1^H NMR (400 MHz, DMSO-d_6_) δ 8.16 (br s, 1H); 2.22 (s, 3H); ^13^C NMR (100 MHz, DMSO-d_6_) δ 189.4, 116.1, 114.2, 58.4, 21.4. Acetylmalononitrile **7** showed 93% purity by QNMR, see Supplementary Material.

For reactions on a half scale, i.e., 9.5 g sodium enolate **8**, all amounts and times were halved, aside from cooling times of 15 min while in an ice-water bath. One DCM and one hexanes slurry was still used, but the amounts of each were halved.

*2-((tert-butyldimethylsilyl)oxy)malononitrile* (**1**). To a 250 mL 24/40 RBF fitted with a 3.0 cm stir bar was added acetylmalononitrile **7** (3.0 g, 27.8 mmol, 1 equiv) followed by water (30 mL, 0.9 M). The flask was topped with a 50 mL addition funnel and placed into an ice-water bath for 15 min. Meanwhile, 10 mL peracetic acid (42 mmol, 1.5 equiv, 32 wt% in acetic acid) was then poured into 20 mL glacial acetic acid (350 mmol, 12.6 equiv). This resulted in a 1.4 M solution of peracetic acid in acetic acid, which was swirled and then poured into the addition funnel and added to the reaction at a rate of 1–2 drops/s (Figure S3A). The reaction lightened in color from light yellow/brown to light yellow. The ice-water bath was removed, and the reaction was stirred for 2 h at rt (it darkens in color some during this time, Figure S3B). At this point, the reaction could be stored in the freezer overnight without a significant loss in yield of the title compound. Alternatively, it can be carried on directly as follows. The stir bar was removed, and the flask was placed on the rotovap with a blast shield in front of the apparatus until no more volatiles were collected, resulting in a yellow oil (water bath not above 30 °C, Figure S3C). The concentration of a solution containing peroxides could potentially result in an explosion; therefore, a blast shield was used every time out of an abundance of caution. To the flask was added the original stir bar and a 90° glass adaptor which was attached to a secondary trap (filled with liquid nitrogen) leading to a manifold, a larger trap (filled with liquid nitrogen), and a high vacuum pump (Figure S3D). A blast shield was added in front of the setup and remained there for the duration of the high vacuum. A room-temperature water bath is placed under the reaction flask to prevent the flask from cooling too much during evaporation. The stirring was started at a low speed (100 rpm), and the manifold valve was slowly opened to the high vacuum. After 2.5 h, a cloudy pale yellow/whitish oil was obtained (Figure S3E), and an aliquot was collected for NMR analysis to confirm the formation of **5** and removal of all volatiles (acetic acid content should be no more than 5%, based on the integration of the peak at 1.90 ppm, or otherwise the yield of the title compound was lower). Peracetic acid is not evident by NMR in DMSO-d_6_ and must decompose such that only peaks of acetic acid are noted. The following analytical data were obtained for hydroxymalononitrile **5**: ^1^H NMR (400 MHz, DMSO-d_6_) δ 6.07; ^13^C NMR (100 MHz, DMSO-d_6_) δ 114.6, 49.8.

To the flask containing crude **5** and the stir bar, we added a septum and balloon, followed by *N,N*-dimethylformamide (28 mL, 1.0 M) via syringe. The reaction flask was placed in an ice-water bath for 15 min and stirred. During this time, imidazole (3.8 g, 55.6 mmol, 2.0 equiv) and *tert*-butyldimethylchlorosilane (6.3 g, 41.7 mmol, 1.5 equiv) are weighed separately, in that order. Then, TBSCl is added in one portion to the reaction flask, followed by imidazole in three portions spaced 1 min apart. The reaction was stirred at 0 °C for 30 min, at which time the reaction progressed in color from yellow to orange. The ice bath was removed, and the reaction was stirred at rt for 30 min (Figure S3F). The reaction was quenched by the addition of 50 mL water followed by 60 mL Et_2_O and was stirred for 30 min. The biphasic mixture was transferred to a 250 mL separatory funnel and the layers were separated. The aqueous layer was extracted with 2 × 40 mL Et_2_O. The combined organic layers were washed with 50 mL satd. aq. NaHCO_3_ followed by 50 mL satd. aq. NaCl, dried over Na_2_SO_4_, and then filtered into a 500 mL 24/40 strawberry flask. Volatiles were removed on the rotovap, which resulted in a crude yellow oil (6.4 g, 20 mmol, 61% purity by ^1^H NMR in CDCl_3_, 72% crude yield, Figure 3a). The crude was purified on a 5.5 cm diameter glass column containing 170 g of silica gel (40–63 μM, 60 Å) and eluted with 1100 mL 3:2 hex:DCM followed by 500 mL 2:3 hex:DCM (75 mL fraction size). The desired product was obtained in fractions 7–23, which were concentrated by rotovap to provide 2-((*tert*-butyldimethylsilyl)oxy)malononitrile (**5**) as a colorless oil (3.9 g, 20 mmol, 71% yield, Figure 3b). The following analytical data were recorded: R*_f_* = 0.24 (3:2 hex:DCM, I_2_ stain, Figure S3G); IR (film) 2957, 2933, 2889, 2862, 2253, 1473, 1259, 1115, 986, 828, 784, 678 cm^−1^; ^1^H NMR (400 MHz, CDCl_3_) δ 5.33 (s, 1H), 0.94 (s, 9H), 0.28 (s, 6H); ^13^C NMR (100 MHz, CDCl_3_) δ 112.3, 50.8, 25.1, 18.0, −5.3; HRMS-ESI calcd. for C_9_H_16_N_2_OSi [2M+Na]^+^: 415.1956; found 415.1953. TBS-MAC **1** showed 97% purity by QNMR; see Supplementary Material.

For reactions on a small scale, i.e., 0.75 g or 1.50 g acetylmalononitrile **7**, all amounts were scaled accordingly. Cooling times of 15 min while in an ice-water bath were maintained. The stirring of **7** with the acids solution was still kept to 2 h at rt. For high vacuum, 2 h was sufficient at 1.50 g and 1.5 h at 0.75 g scale. Crude **5** was always checked by ^1^H and ^13^C NMR for purity before proceeding with the protection step. After the addition of imidazole and TBSCl, the times of 0 °C for 30 min and rt for 30 min were still maintained.

## 4. Conclusions

This paper describes the scalable and detailed preparation of TBS-MAC **1** from acetylmalononitrile **7**, such that this valuable masked acyl synthon can be accessed reliably in 60–70% yields at three synthetically useful scales of 0.75 g, 1.5 g, or 3.0 g. TBS-MAC **1** can be stored indefinitely in the freezer and in the refrigerator while being used. Moreover, storage of the intermediate hydroxymalononitrile **5** in the acidic solution overnight allows for breaking up an otherwise long reaction over two days if desired. With a reliable synthesis of **5**, other valuable masked acyl synthons can also be accessed. We also described the detailed two-step synthesis of acetylmalononitrile **7** from sodium enolate **8** and, ultimately, malononitrile **1** on either 5 g or 10 g scales. Both sodium enolate **8** and acetylmalononitrile **7** are bench-stable solids and can be stored for over a year without any special precautions. Our detailed synthetic procedures provide sodium enolate **8** and help to fill in some gaps noted in the literature for the synthesis of **7** and **1**, thereby increasing the accessibility of these valuable masked acyl cyanide (MAC) reagents.

## Figures and Tables

**Table 1:** Conversion of malononitrile **6** to sodium enolate **8**.

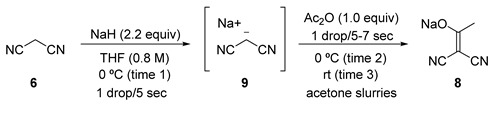					
Entry	Scale	Time 1	Times 2, 3	Acetone Slurry	Yield
1	5 g	30 min	15 min, 15 min	2 × 100 mL	9.5 g, 97%
2	10 g	1 h	30 min, 30 min	2 × 200 mL	17.3 g, 88%
3	10 g	1 h	30 min, 30 min	3 × 100 mL	13.8 g, 70%
4	10 g	1 h	30 min, 30 min	3 × 200 mL	18.7 g, 95%
5	10 g	1 h	30 min, 30 min	4 × 200 mL	19.2 g, 98%

**Table 2:** Conversion of sodium enolate **8** to acetylmalononitrile **7**.

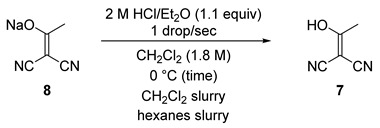					
Entry	Scale	Time	CH_2_Cl_2_ Slurry	Hexanes Slurry	Yield
1	9.5 g	15 min	1 × 50 mL	1 × 50 mL	6.8 g, 86%
2	19.2 g	30 min	1 × 100 mL	1 × 100 mL	13.6 g, 85%

**Table 3:** Conversion of acetylmalononitrile **8** to TBS-MAC **1**.

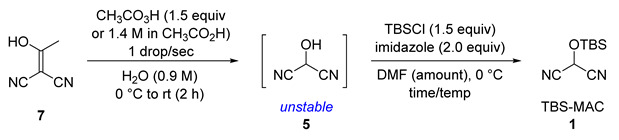					
Entry	Scale	DMF	Time/Temp ^[a]^	Crude (g, Yield) ^[c]^	Purified Yield ^[d]^
1	0.75 g	0.5 M	30 min 0 °C, 30 min rt	0.83 g, 61%	---
2	0.75 g	0.5 M	30 min 0 °C, 12 h rt	0.85 g, 63%	---
3	0.75 g	1 M	30 min 0 °C, 30 min rt	0.92 g, 68%	0.81 g, 60%
4	0.75 g	1 M	1 h rt	0.86 g, 63%	---
5	0.75 g	1 M	30 min 0 °C, 30 min rt ^[b]^	0.66 g, 48%	---
6	1.5 g	1 M	30 min 0 °C, 30 min rt	1.94 g, 71%	1.84 g, 68%
7	3.0 g	1 M	30 min 0 °C, 30 min rt	3.91 g, 72%	3.90 g, 71%

^[a]^ DMF was added to the reaction flask containing crude **5** and cooled to 0 °C for 15 min. Then TBSCl was added in one portion, followed immediately by imidazole in 3 portions. Time and temperature entries correspond to stirring after the addition of imidazole. ^[b]^ Entry 5 contained the reverse order: imidazole first in one portion, then TBSCl in three portions. ^[c]^ Crude amounts and yields for **1** were recorded after ^1^H NMR and accounted for impurities such as remaining DMF, EtOAc (from extraction), and the byproduct TBS-OH (not shown). For example, in entry 1, the crude amount was 1.36 g. However, taking into account the byproduct and impurities resulted in 0.83 g of **1**, which corresponds to a 61% crude yield. Using this method, we did not purify each entry as the crude yields were accurate indicators of the reaction’s success. ^[d]^ Purified yield corresponds to **1** after column chromatography on SiO_2_.

## Data Availability

The analytical data obtained in this study are available within the Materials and Methods section, and copies of spectra and pictures of reaction steps are available in the Supplementary Material.

## Supplementary Materials

The following supporting information can be downloaded at: https://www.mdpi.com/article/10.3390/molecules28135087/s1, IR spectra, NMR spectra (^1^H and ^13^C and QNMR), and pictures corresponding to the various steps in the syntheses.

## Sample Availability

Samples of the compounds can be obtained by following the detailed procedures described therein. Acetylmalononitrile **7** and TBS-MAC **1** can be purchased in small amounts through chemical vendors.
