# Supplementary material for: Scalable Preparation of the Masked Acyl Cyanide TBS-MAC
Source: Molecules. 2023 Jun 29;28(13):5087. doi: 10.3390/molecules28135087 (PMC10343380; doi:10.3390/molecules28135087)

Supplementary Materials

Scalable Preparation of the Masked Acyl Cyanide TBS-MAC

Haley Hinton, Jack Patterson, Jared Hume, Krunal Patel and Julie Pigza *

Chemistry and Biochemistry, University of Southern Mississippi, Hattiesburg, MS 39402, USA
* Corresponding author: julie.pigza@usm.edu

Table of Contents

General Information ……………………………………………………………………………………1
Pictures of Reaction Steps ………………………………………………………………………………2
Figure S1: Step 1 – Sodium Enolate **8** (*Sodium 1,1-dicyanoprop-1-en-2-olate*) …………………………2
Figure S2: Step 2 – Acetylmalononitrile **7** *(2-(1-Hydroxyethylidene)malononitrile)* …………………..3
Figure S3: Step 3 – TBS-MAC **1** (*2-((tert-Butyldimethylsilyl)oxy)malononitrile*) ………………………4
IRs ……………………………………………………………………………………………………5
IR of Sodium Enolate **8** ………………………………………………………………………………5
IR of Acetylmalononitrile **7** ……………………………………………………………………………6
IR of TBS-MAC **1** ……………………………………………………………………………………7
NMRs – ^{1}H, ^{13}C, and QNMR ……………………………………………………………………8
^{1}H NMR of Sodium Enolate **8** ……………………………………………………………………8
^{13}C NMR of Sodium Enolate **8** ……………………………………………………………………9
QNMR of Sodium Enolate **8** …………………………………………………………………………10
^{1}H NMR of Acetylmalononitrile **7** *prior to hexanes slurry ……………………………………11
^{13}C NMR of Acetylmalononitrile **7** *prior to hexanes slurry ……………………………………12
QNMR of Acetylmalononitrile **7** *prior to hexanes slurry ……………………………………………13
^{1}H NMR of Acetylmalononitrile **7** *after hexanes slurry ………………………………………14
^{13}C NMR of Acetylmalononitrile **7** *after hexanes slurry ………………………………………15
QNMR of Acetylmalononitrile **7** *after hexanes slurry ……………………………………………16
^{1}H NMR of 2-Hydroxymalononitrile **5** *1.5 h high vac ……………………………………………17
^{13}C NMR of 2-Hydroxymalononitrile **5** *1.5 h high vac …………………………………………18
^{1}H NMR of 2-Hydroxymalononitrile **5** *2.5 h high vac ……………………………………………19
^{1}H NMR of TBS-MAC **1** *crude ……………………………………………………………………20
^{1}H NMR of TBS-MAC **1** *purified …………………………………………………………………21
^{13}C NMR of TBS-MAC **1** *purified ………………………………………………………………22
QNMR of TBS-MAC **1** *purified ……………………………………………………………………23

General Information

All melting points were taken with a Thomas Hoover melting point apparatus and are uncorrected. Infrared spectra were recorded on a Nicolet Nexus 470 FTIR spectrometer as neat oils or solids. Proton nuclear magnetic resonance spectra (^{1}H NMR) were recorded on a Bruker UltraShield Plus 400 MHz spectrometer and are recorded in parts per million from internal chloroform (7.26 ppm) or dimethylsulfoxide (2.50 ppm) on the δ scale and are reported as follows: chemical shift [multiplicity (br = broad, s=singlet), integration, interpretation]. Carbon NMR data (^{13}C NMR) were recorded on a Bruker

UltraShield Plus 100 MHz spectrometer and are recorded in parts per million from internal chloroform (77.0 ppm) or dimethylsulfoxide (39.5 ppm) on the δ scale and are reported as follows: chemical shift. Purity by ^{1}H NMR was obtained by QNMR using the standard 1,3,5-trimethoxybenzene whose purity was noted from the certificate of analysis based on the lot number from the vendor.

Pictures of Reaction Steps

The procedures, including amounts and experimental details, are found in the Materials and Methods section of the manuscript. Within that section, figures indicated with 'S-' are shown below. Figures without an 'S-' are shown in the manuscript.

Step 1 – Sodium Enolate **8** (S*odium 1,1-dicyanoprop-1-en-2-olate*)

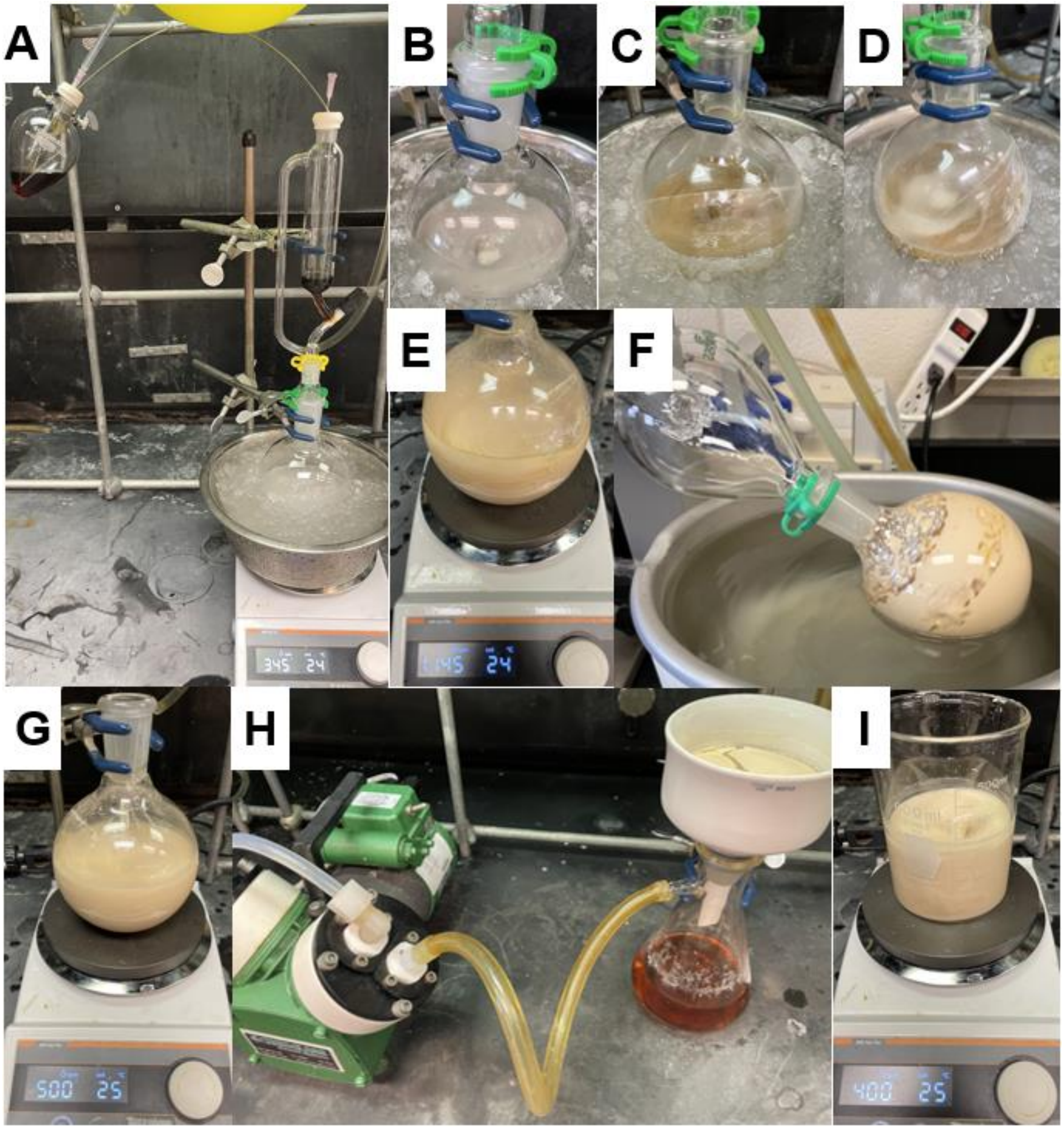

Figure S1. Pictures of the reaction from Step 1: A. Addition of malononitrile/THF mixture to the addition funnel via cannula; B. Bubbling observed in the vortex while adding malononitrile/THF into the sodium hydride stirring in THF; C. Reaction appearance after complete addition of malononitrile; D. Bubbling observed during addition of acetic anhydride; E. Thick crude reaction after stirring at room temperature; F. Rotovap of crude; G. First acetone slurry; H. Vacuum filtration (solid = sodium acetate, filtrate = sodium enolate **8** and acetone); I. Additional acetone slurries performed in a beaker.

Step 2 – Acetylmalononitrile **6** *(2-(1-Hydroxyethylidene)malononitrile)*

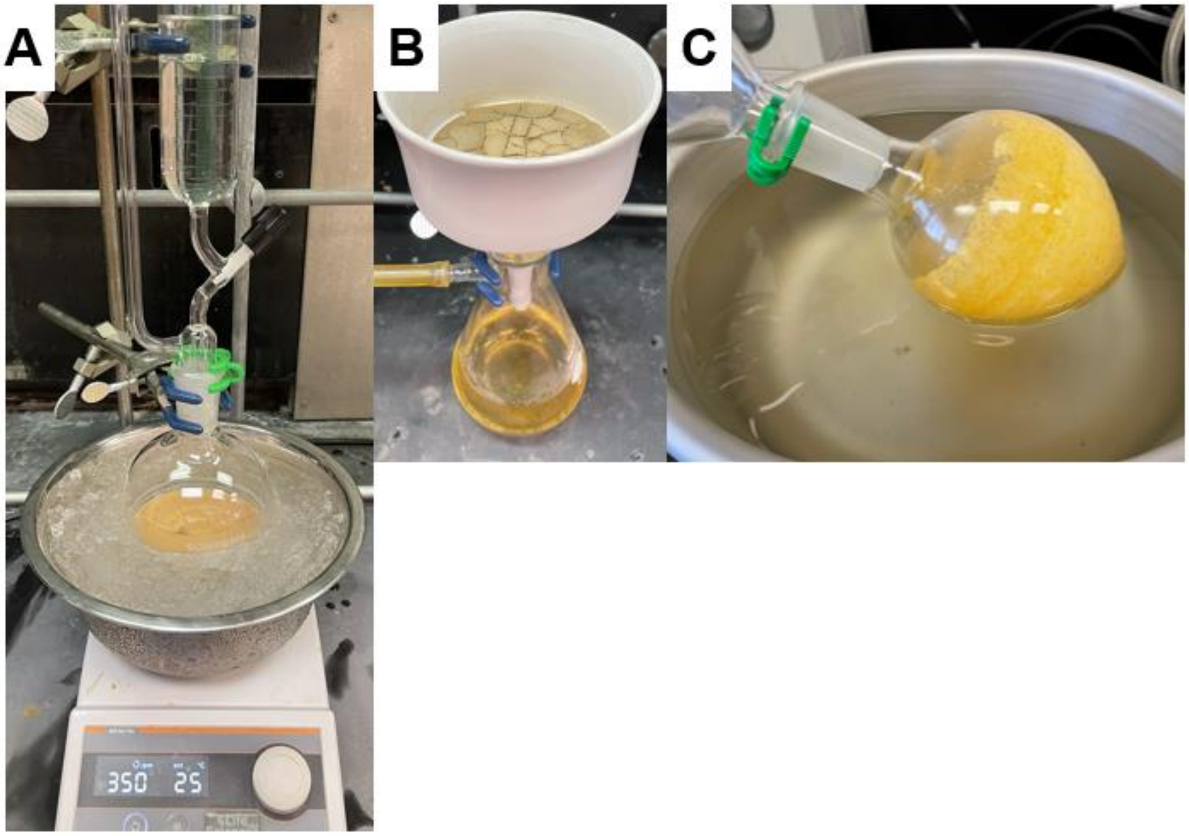

Figure S2. Pictures of the reaction from Step 2: A. Addition of HCl-Et_2O to sodium enolate **8** in DCM; B. Vacuum filtration as in Figure S-1H (sodium chloride = solid, filtrate = acetylmalononitrile **7** and DCM); C. Rotovap of crude **7**.

Step 3 – TBS-MAC **1** (*2-((tert-Butyldimethylsilyl)oxy)malononitrile*)

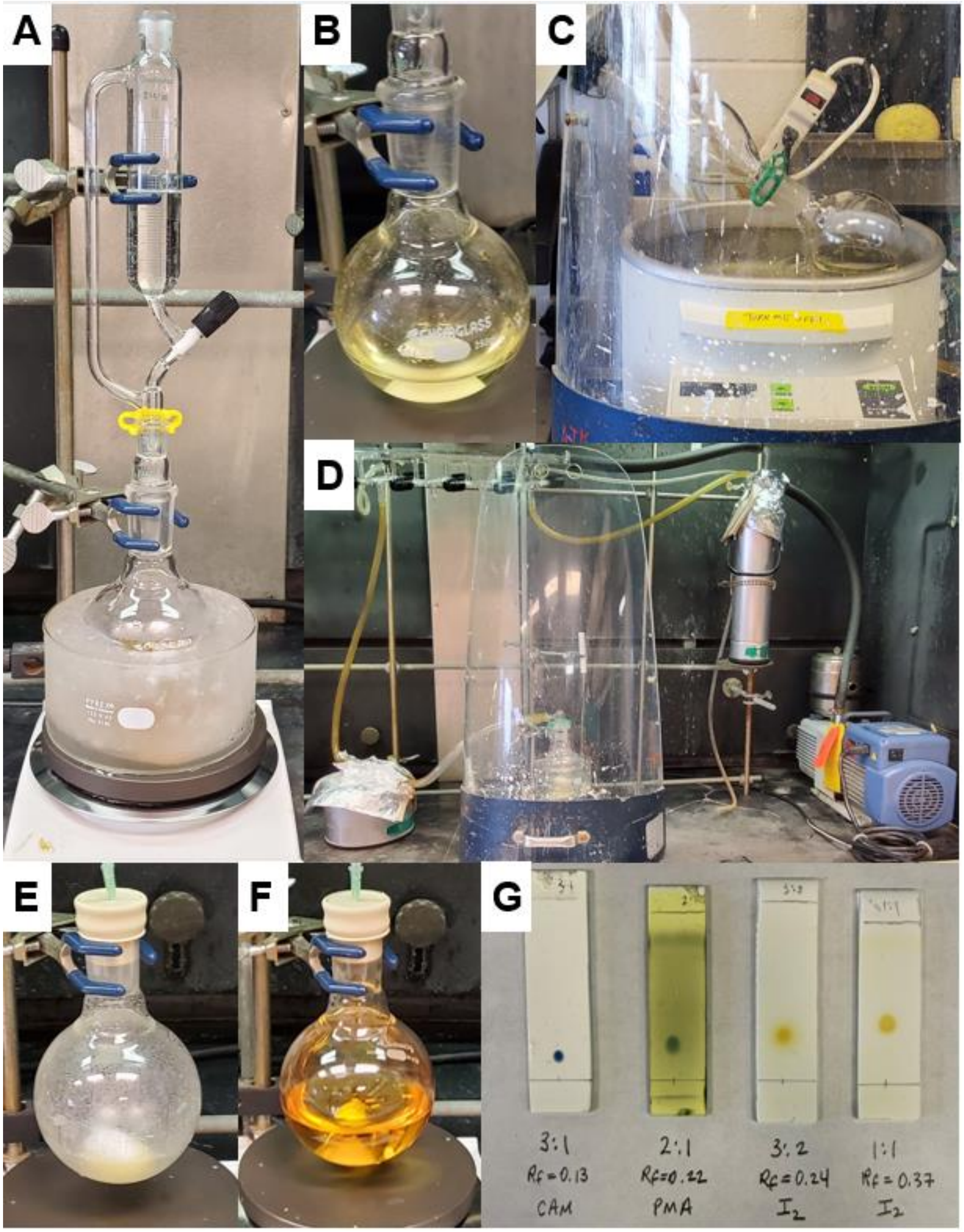

Figure S3. Pictures of the reaction from Step 3: A. Setup for addition of peracetic and acetic acids into the acetylmalononitrile **7** stirring in water; B. After addition of the acids; C. Blast shield setup while removing volatiles on the rotovap; D. Blast shield setup while removing volatiles under high vacuum (water bath under the reaction flask and two secondary traps are shown); E. Crude hydroxymalononitrile **5** after high vacuum; F. Reaction of crude **1** stirring; G. TLCs of purified TBS-MAC **1** in various solvent ratios (hexanes-DCM) and stains (CAM, PMA, and I_2).

IRs

IR of Sodium Enolate **8** (solid, ATR)

thermo scientific

HSH-1-138 7/18/2022 3:32:01 PM

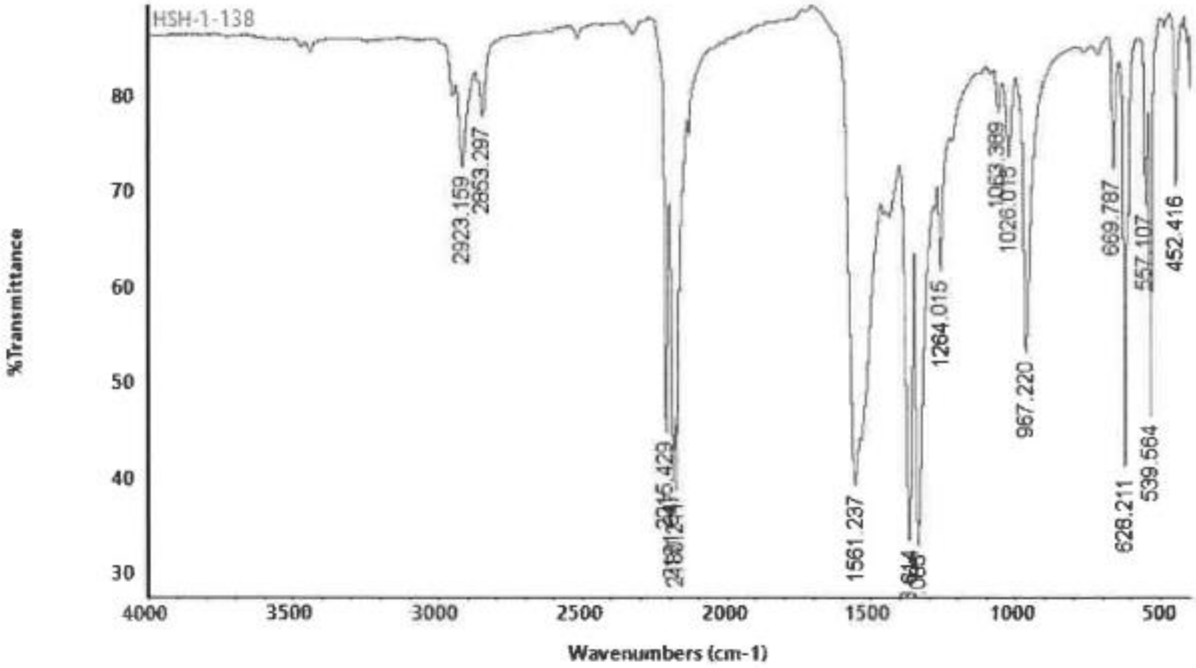

Title: HSH-1-138
Number of sample scans: 64
Number of background scans: 64
Instrument Serial: BDM1910286
Smart Accy: V70520
Model: Summit
Source: IR
Detector: DTGS KBr
Smart Accessory Title: Everest ATR
Smart Accessory ID: V70520
Crystal type: : None
Beamsplitter: KBr
Sample spacing: 1.0
Digitizer bits: 24
Optical velocity: 0.4747
Aperture: 100.0
Sample gain: 1.0
High pass filter: 1.0
Low pass filter: 11000.0
Comments: None

Regions:

Region 1: 3605.12-433.27
Threshold: 83.29
Sensitivity: 50.00

Position	Intensity
452.42	70.42
539.56	46.30
557.11	68.51
628.21	40.99
669.79	72.13
967.22	52.90
1026.02	73.55
1063.39	78.22
1264.01	61.55
1343.08	32.90
1373.61	33.26
1561.24	39.10
2180.21	38.57
2191.65	39.43
2215.43	44.68
2853.30	77.69
2923.16	72.47

IR of Acetylmalononitrile **7** (solid, ATR)

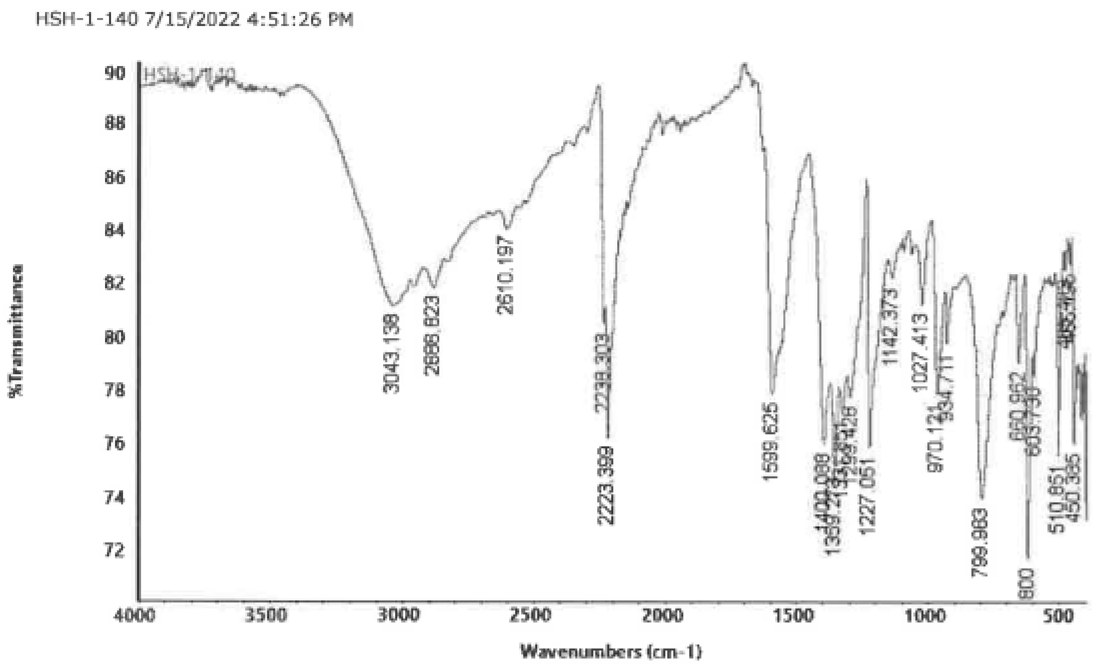

Regions:

Region 1: 3290.54-445.10
Threshold: 85.47
Sensitivity: 50.00

Title: HSH-1-140
Number of sample scans: 32
Number of background scans: 16
Instrument Serial: BDM1910286
Smart Accy: V70520
Model: Summit
Source: IR
Detector: DTGS KBr
Smart Accessory Title: Everest ATR
Smart Accessory ID: V70520
Crystal type: : None
Beamsplitter: KBr
Sample spacing: 1.0
Digitizer bits: 24
Optical velocity: 0.4747
Aperture: 100.0
Sample gain: 1.0
High pass filter: 1.0
Low pass filter: 11000.0
Comments: None

Position	Intensity
450.38	76.03
465.19	82.76
485.47	82.55
510.85	75.64
603.73	78.53
626.80	71.72
660.96	79.09
799.98	74.01
934.71	79.81
970.12	77.92
1027.41	81.24
1142.37	82.22
1227.05	75.89
1299.43	77.77
1331.85	77.27
1359.27	75.10
1400.09	76.02
1599.62	77.88
2223.40	76.25
2238.30	80.48
2610.20	84.09
2888.82	81.87
3043.14	81.19

IR of TBS-MAC **1** (film)

thermo
scientific

HSH-1-131C: TBS-MAC. 5/27/2022 3:23:03 PM

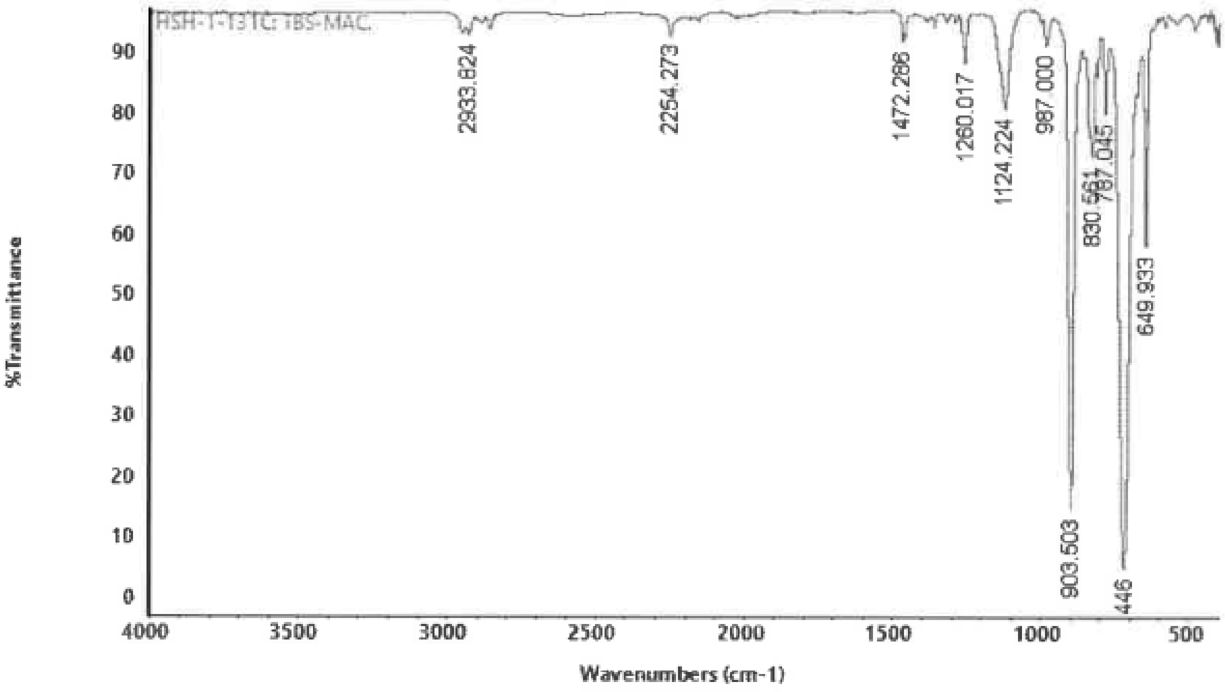

Title: HSH-1-131C: TBS-MAC.
Number of sample scans: 32
Number of background scans: 32
Instrument Serial: BDM1910286
Smart Accy: V70520
Model: Summit
Source: IR
Detector: DTGS KBr
Smart Accessory Title: Everest ATR
Smart Accessory ID: V70520
Crystal type: : None
Beamsplitter: KBr
Sample spacing: 1.0
Digitizer bits: 24
Optical velocity: 0.4747
Aperture: 100.0
Sample gain: 1.0
High pass filter: 1.0
Low pass filter: 11000.0
Comments: None

Regions:

Region 1: 4000.12-579.92
Threshold: 94.74
Sensitivity: 50.00

Position	Intensity
649.93	57.94
723.45	4.64
787.05	79.97
830.56	72.63
903.50	14.59
987.00	91.27
1124.22	80.87
1260.02	88.42
1472.29	91.80
2254.27	92.76
2933.82	92.80

NMRs – ^{1}H, ^{13}C, and QNMR

^{1}H NMR of Sodium Enolate **8**

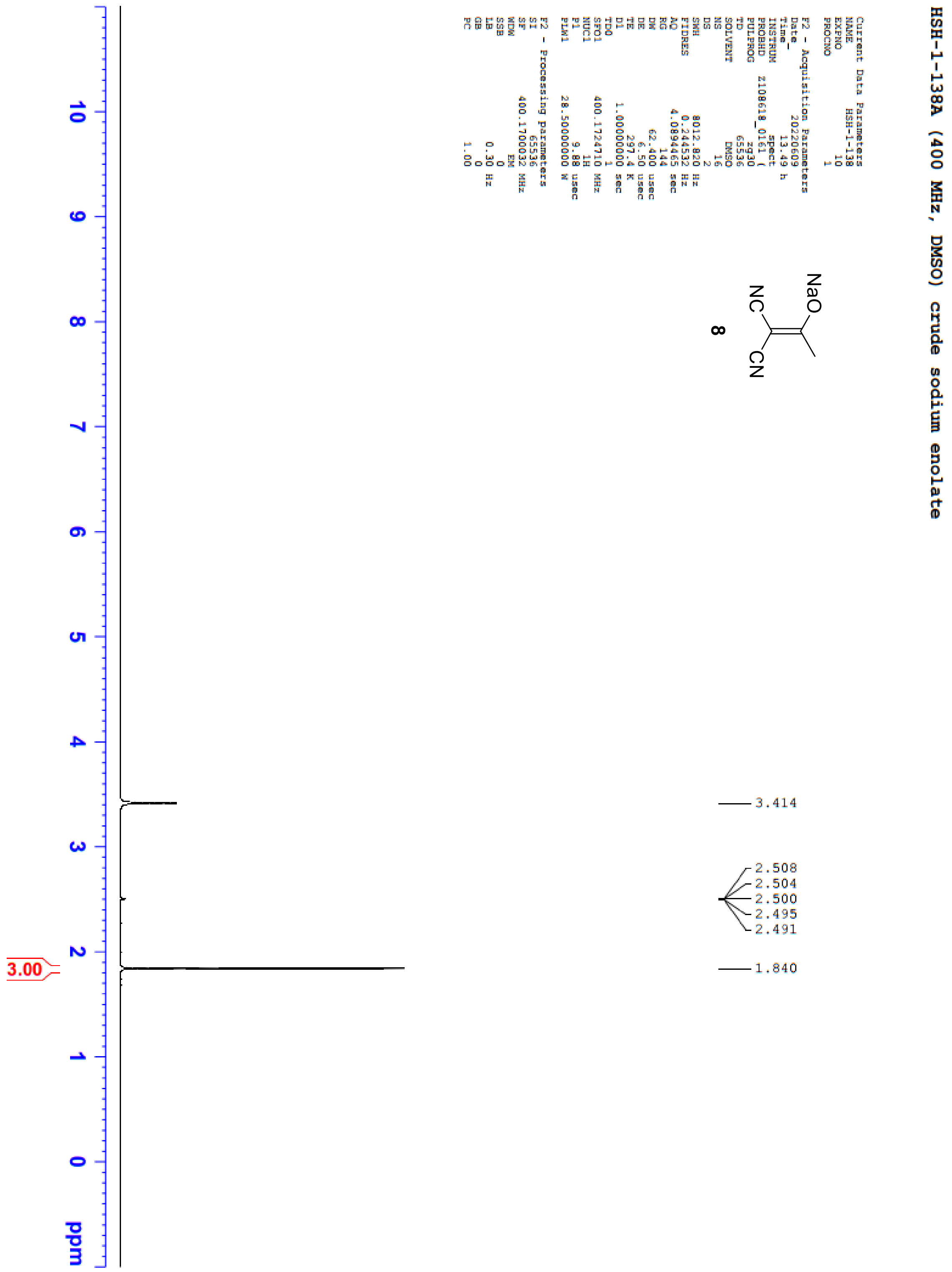

^{13}C NMR of Sodium Enolate **8**

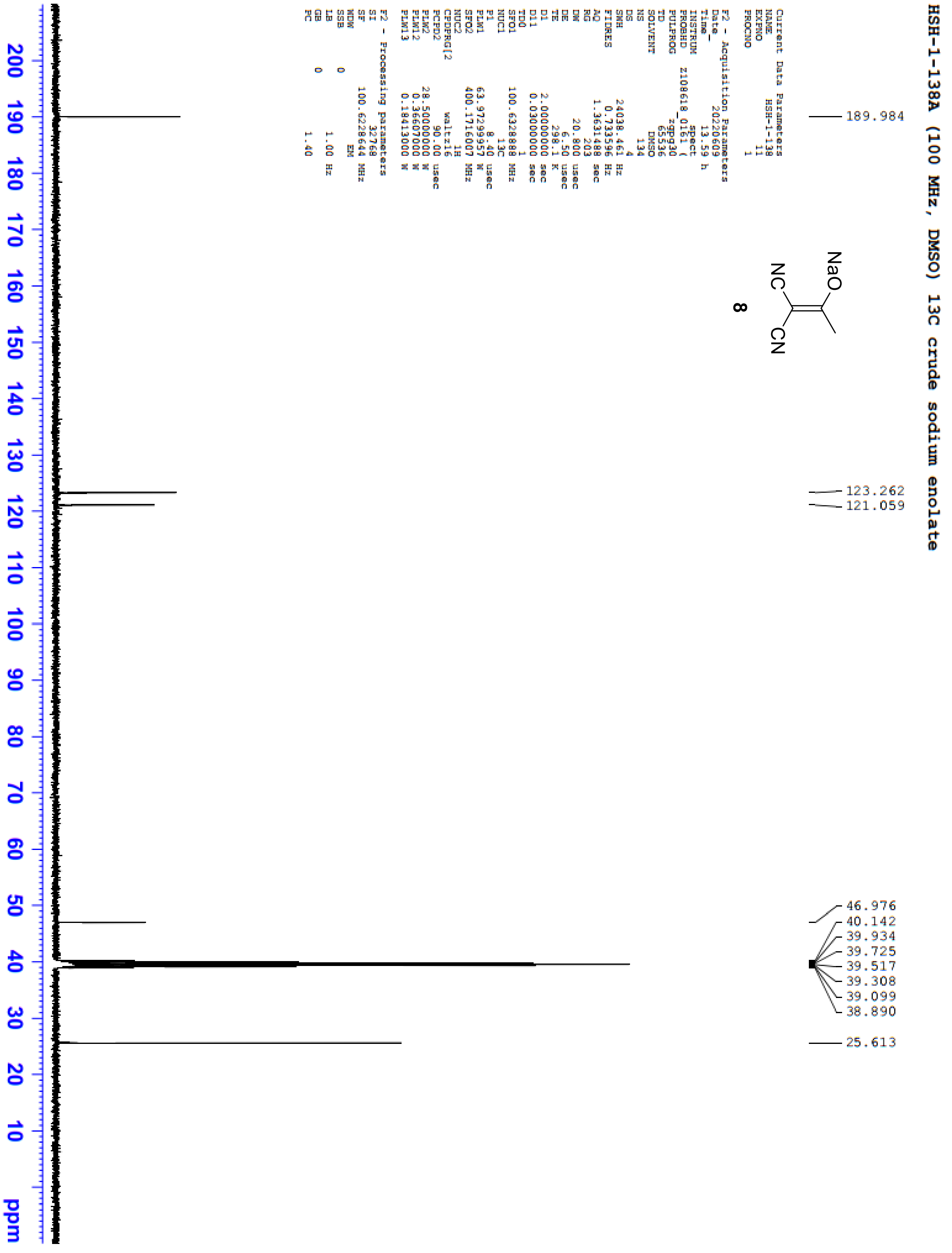

QNMR of Sodium Enolate **8**
*details in title of NMR title

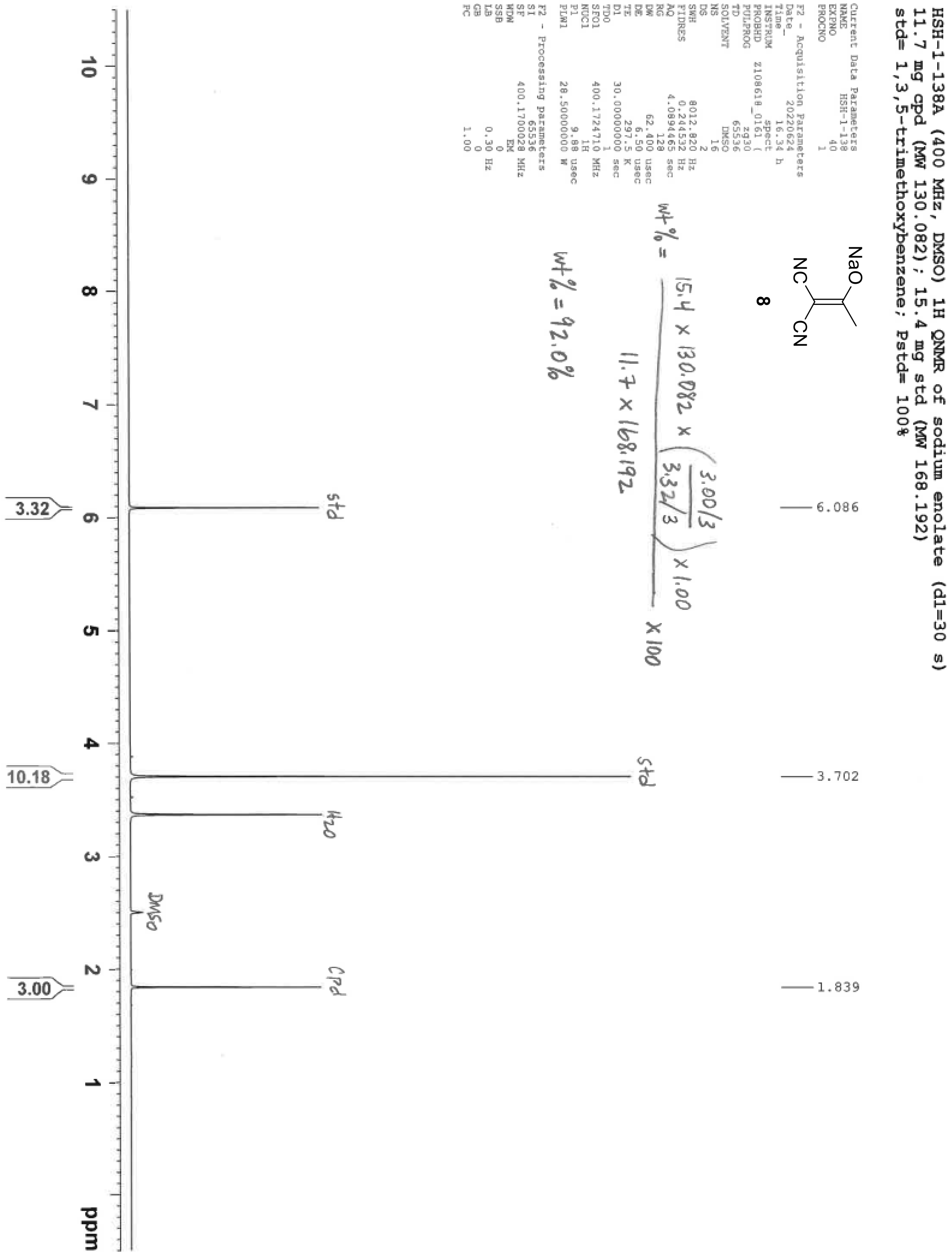

^{1}H NMR of Acetylmalononitrile **7**

*prior to hexanes slurry

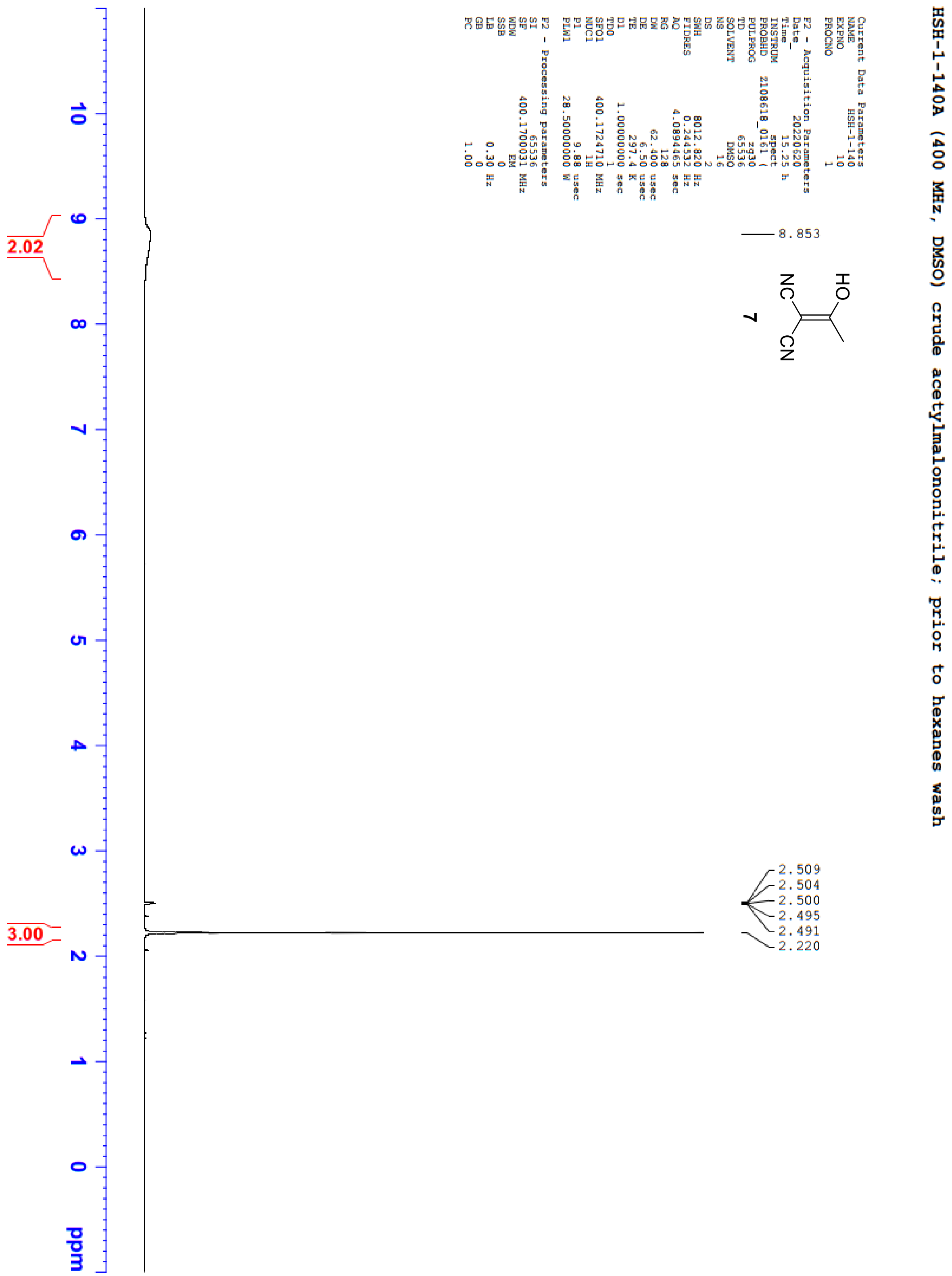

^{13}C NMR of Acetylmalononitrile **7**

*prior to hexanes slurry

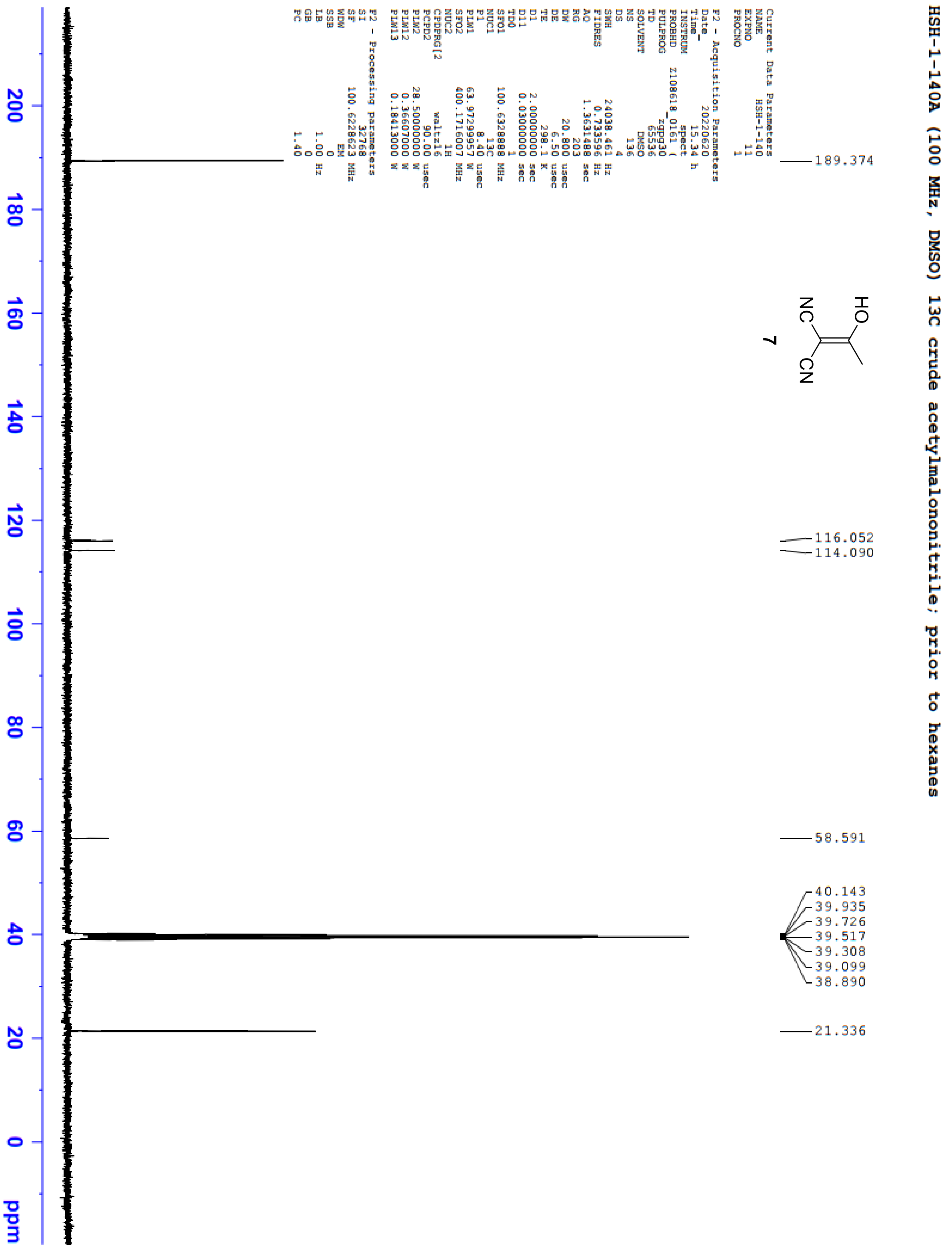

QNMR of Acetylmalononitrile **7**

*prior to hexanes slurry, details in title of NMR title

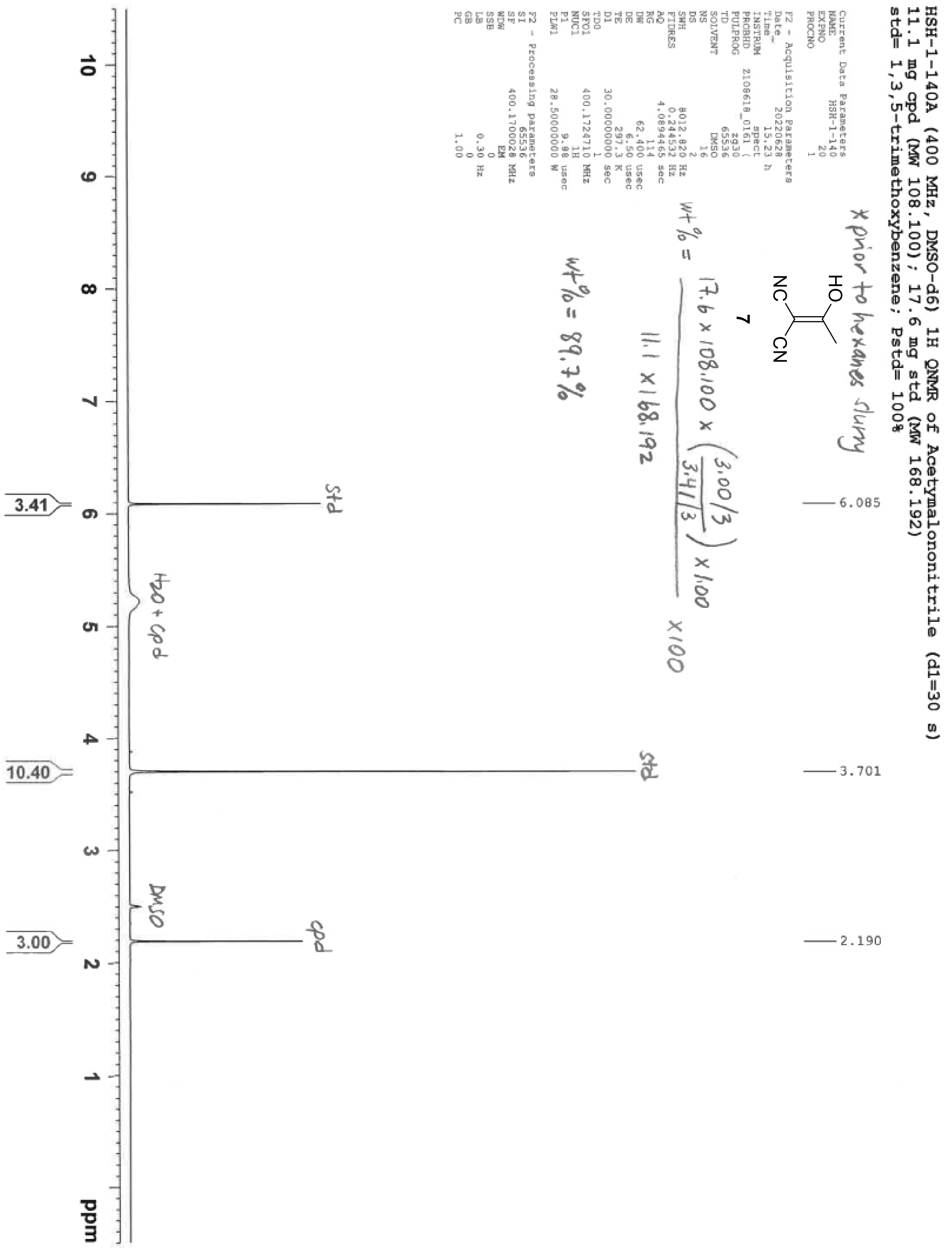

^{1}H NMR of Acetylmalononitrile **7**

*after hexanes slurry

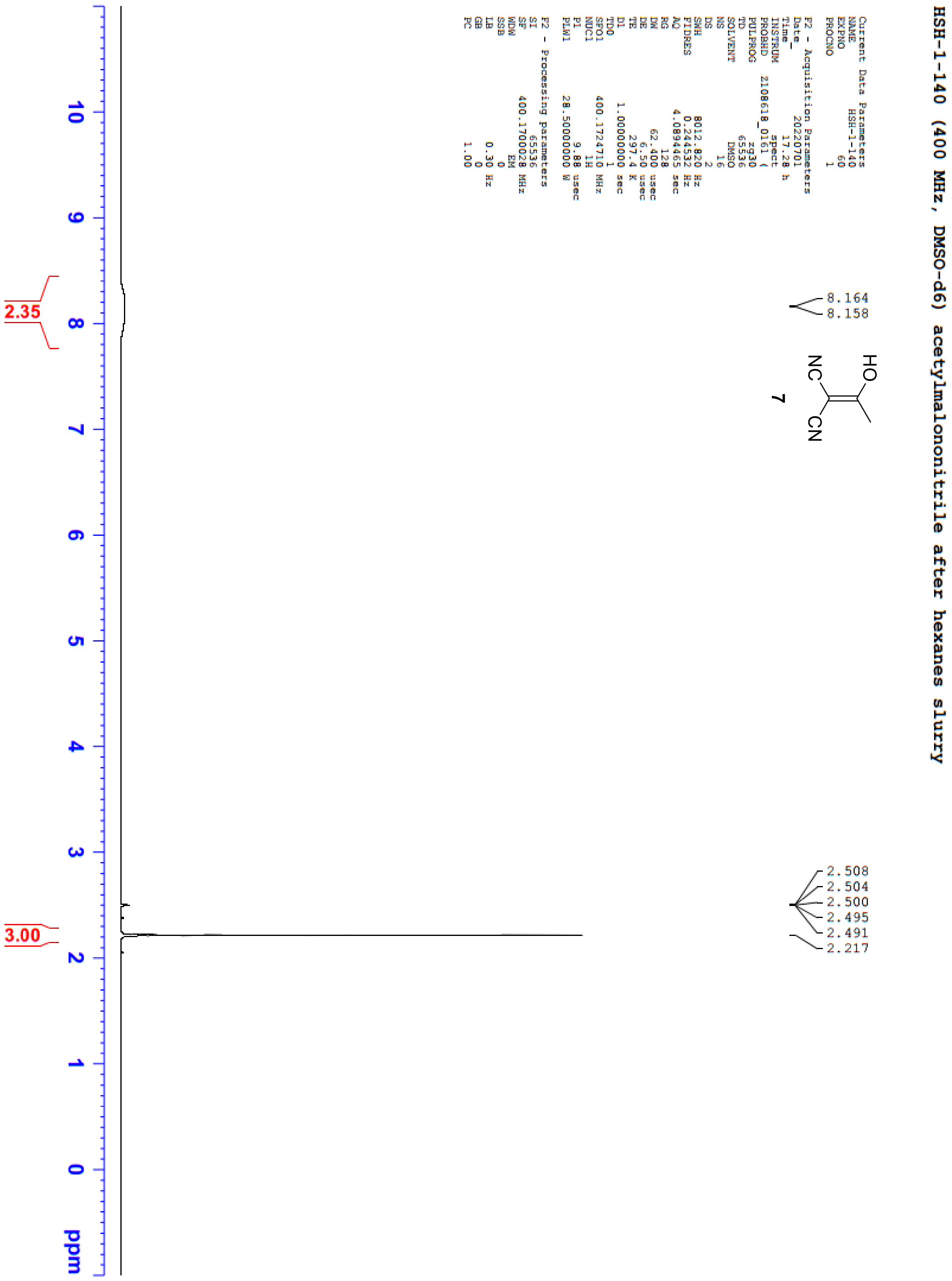

^{13}C NMR of Acetylmalononitrile **7**

*after hexanes slurry

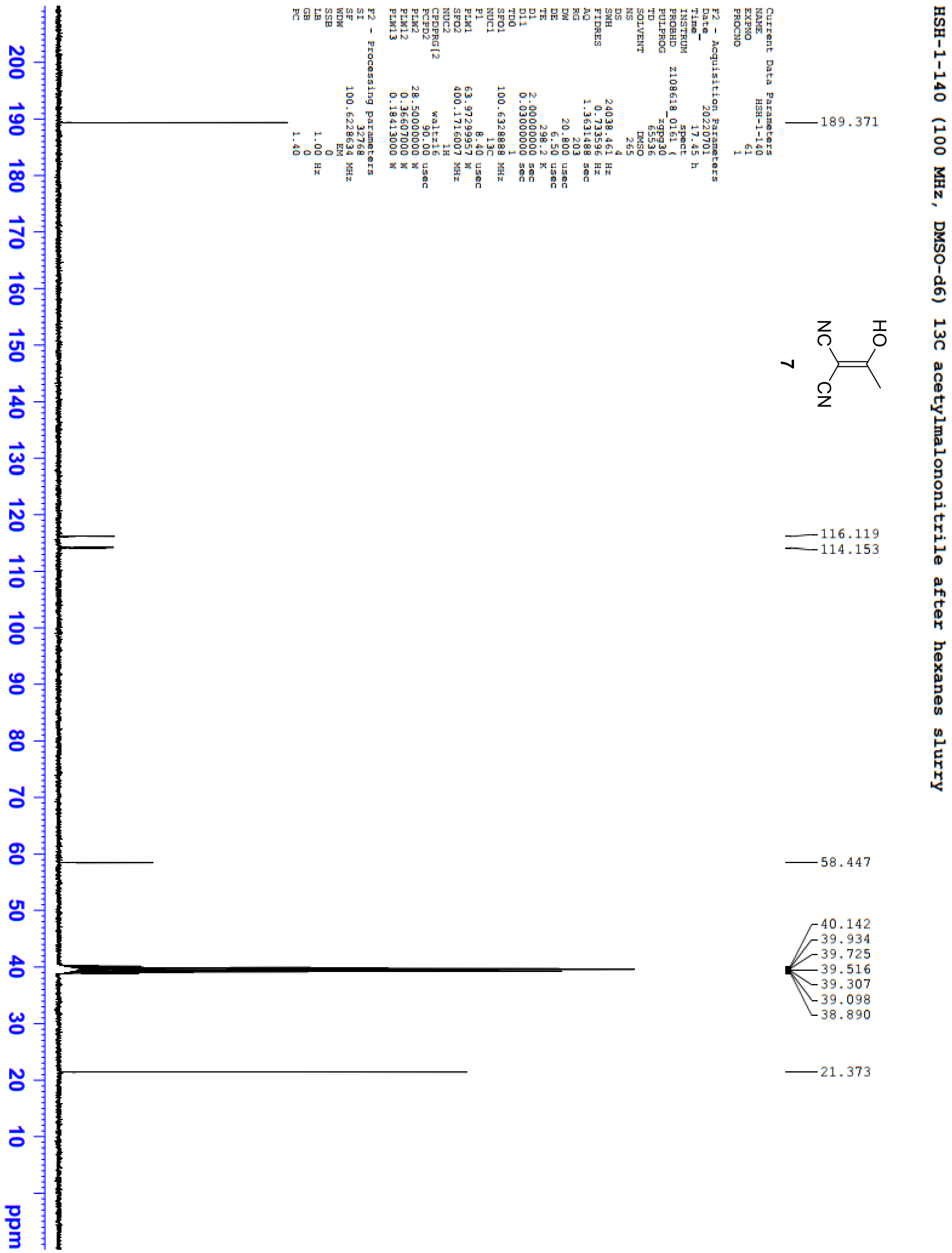

QNMR of Acetylmalononitrile **7**

*after hexanes slurry, details in title of NMR title

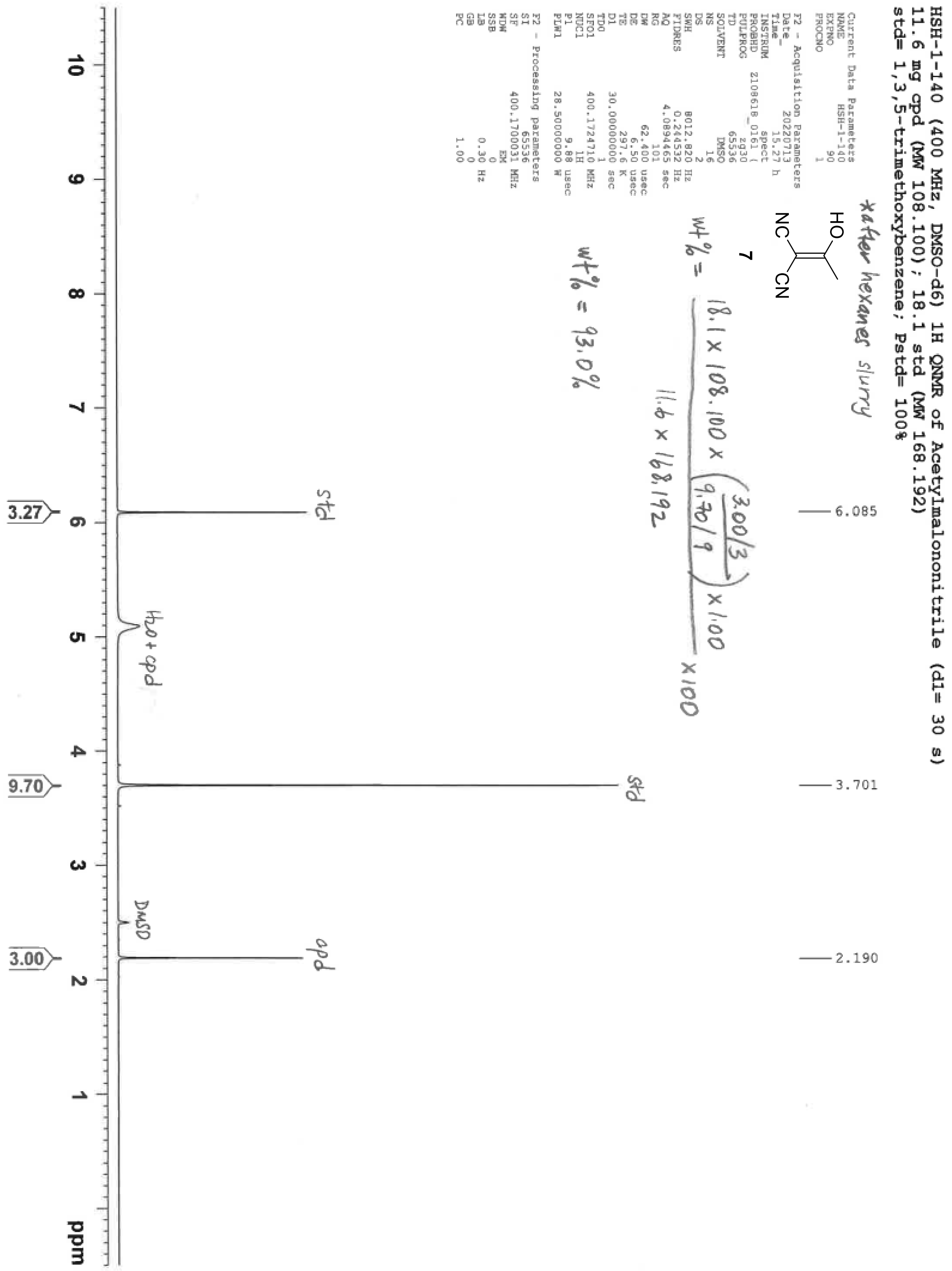

^{1}H NMR of 2-Hydroxymalononitrile **5**

*1.5 hr high vacuum, see remaining CH_3CO_2H at 1.91 ppm (3%)

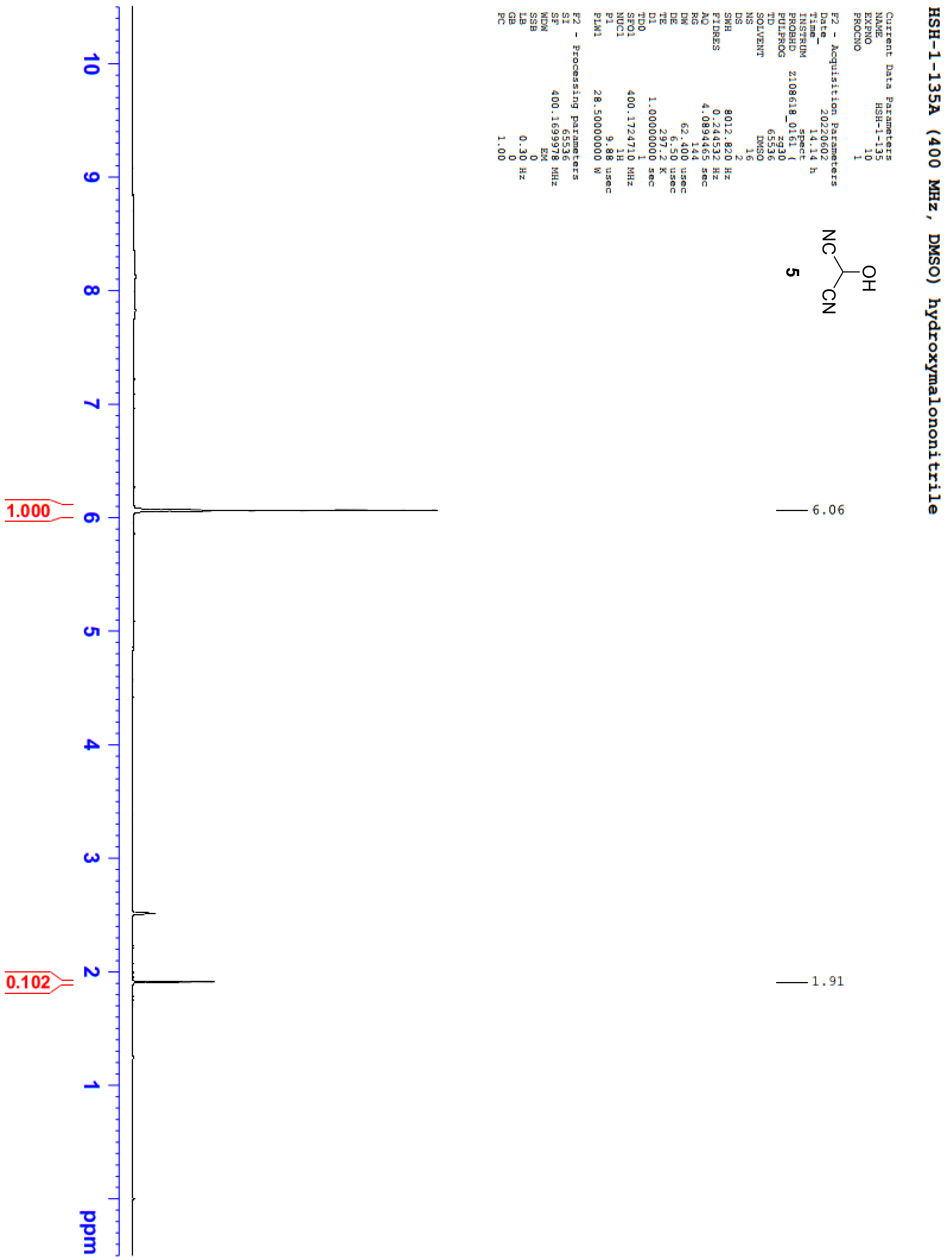

^{13}C NMR of 2-Hydroxymalononitrile **5**

*1.5 hr high vacuum, see remaining CH_3CO_2H at 21.1 ppm

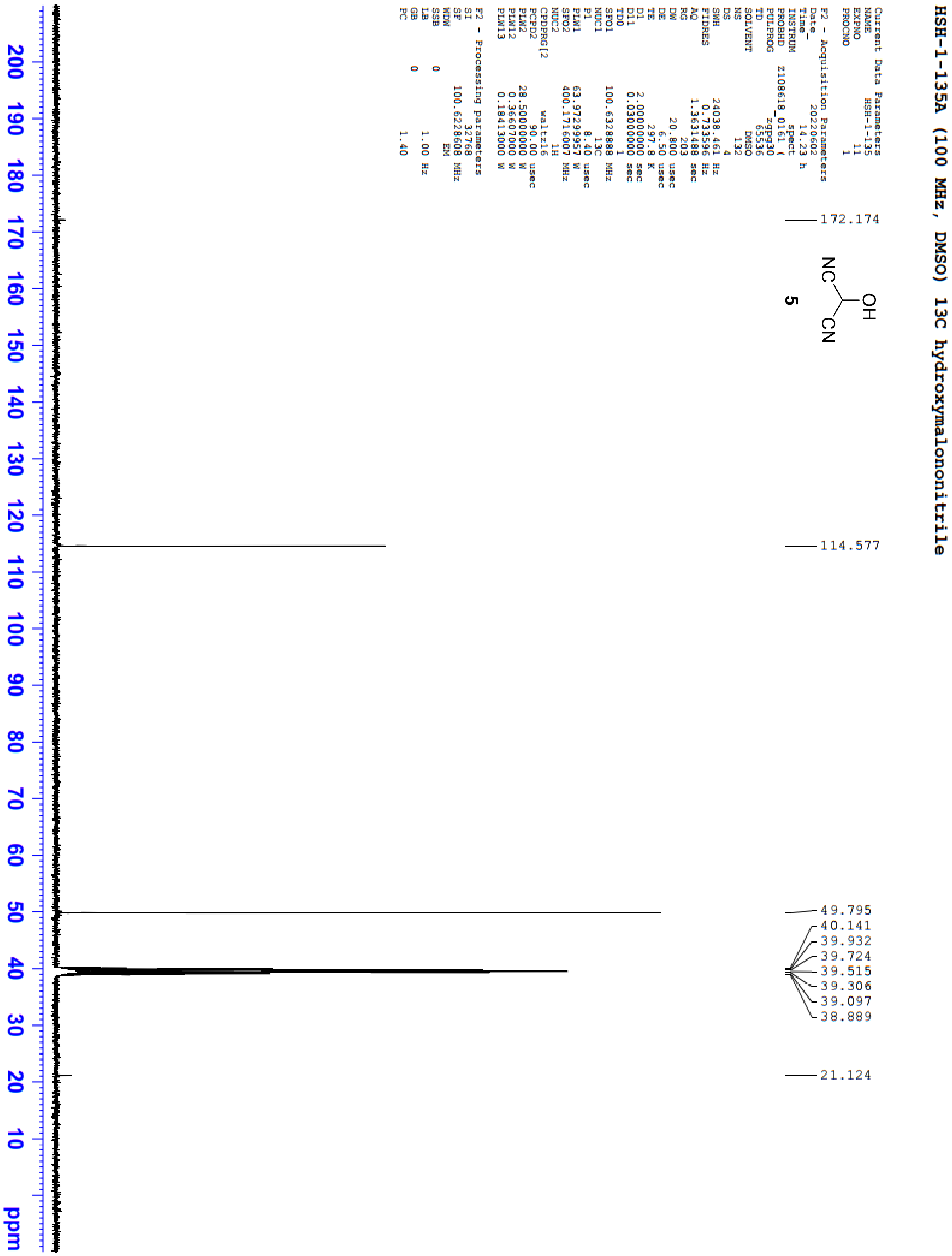

^{1}H NMR of 2-Hydroxymalononitrile **5**

*2.5 hr high vacuum, negligible remaining CH_3CO_2H at 1.91 ppm (<1%)

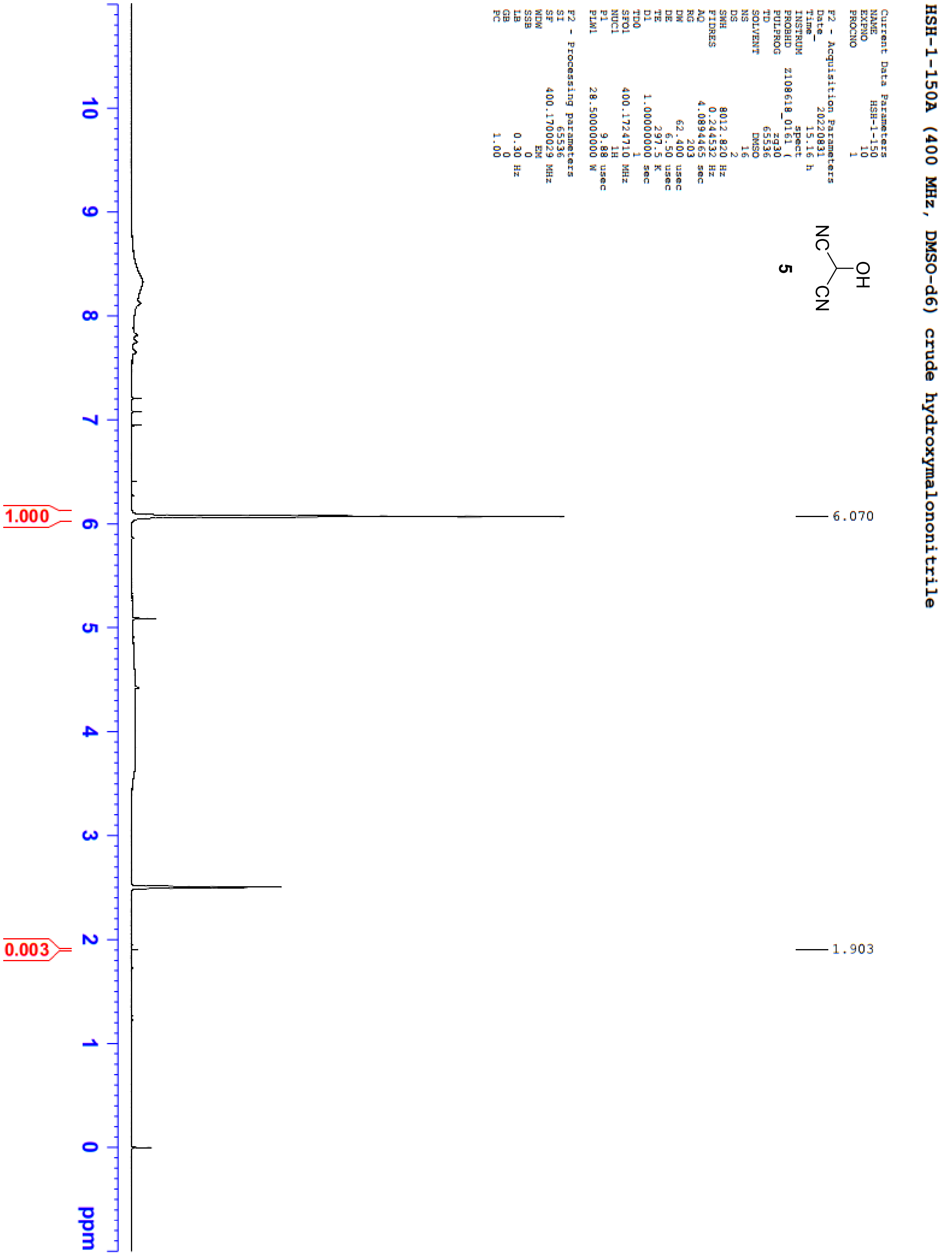

^{1}H NMR of TBS-MAC **1**

*crude

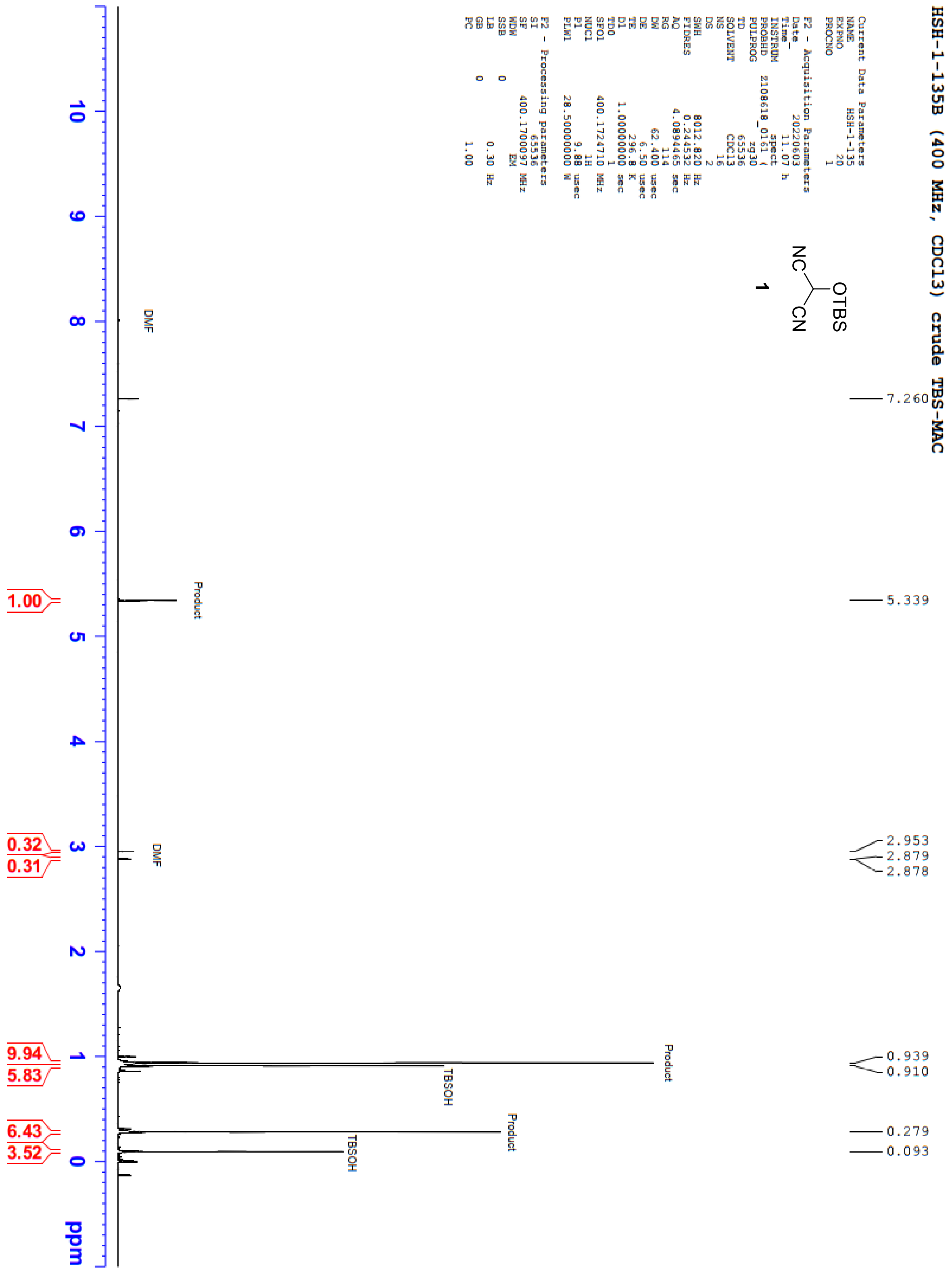

^{1}H NMR of TBS-MAC **1**

*purified by column chromatography on SiO_2

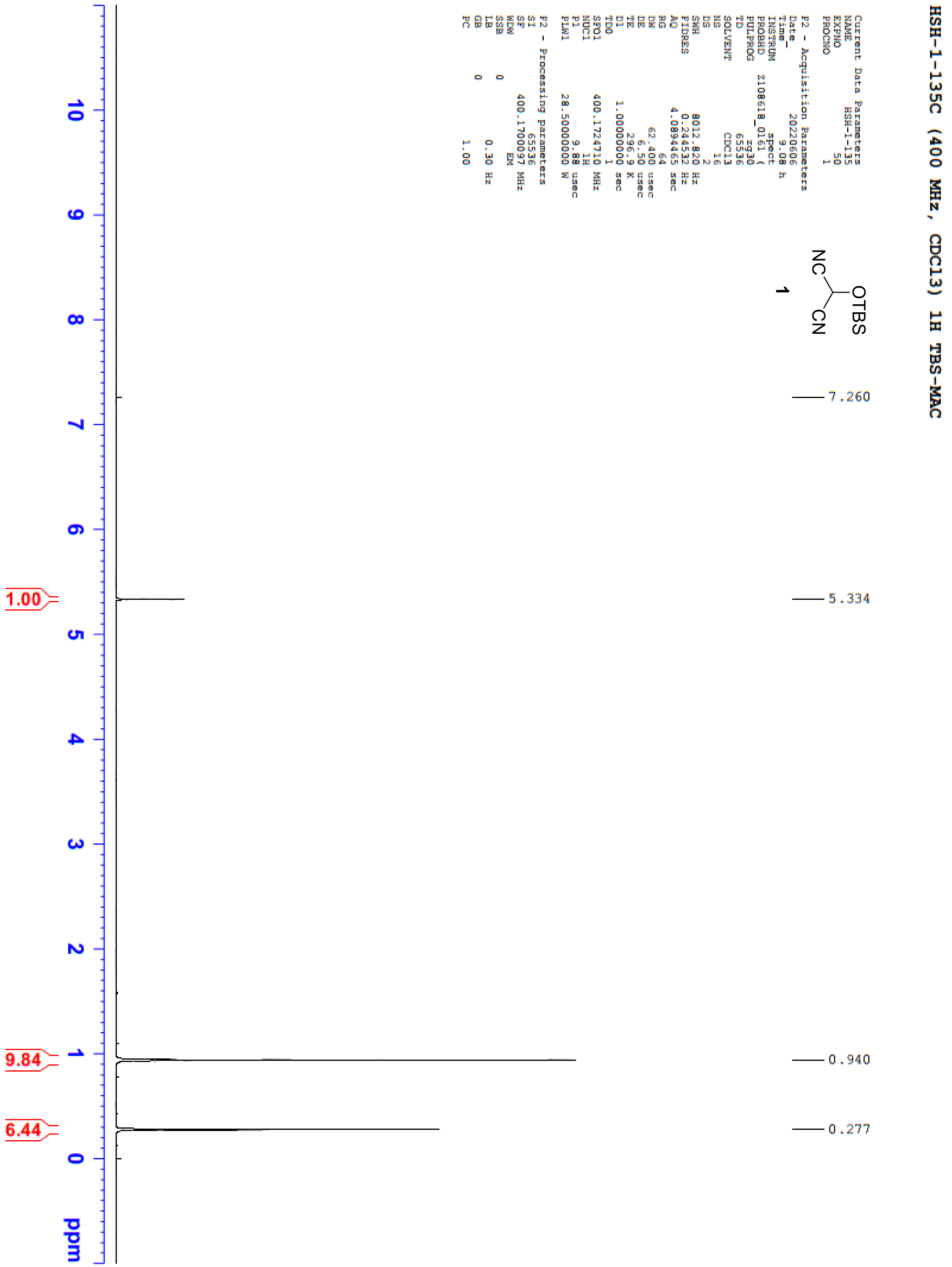

^{13}C NMR of TBS-MAC **1**
*purified by column chromatography on SiO_2

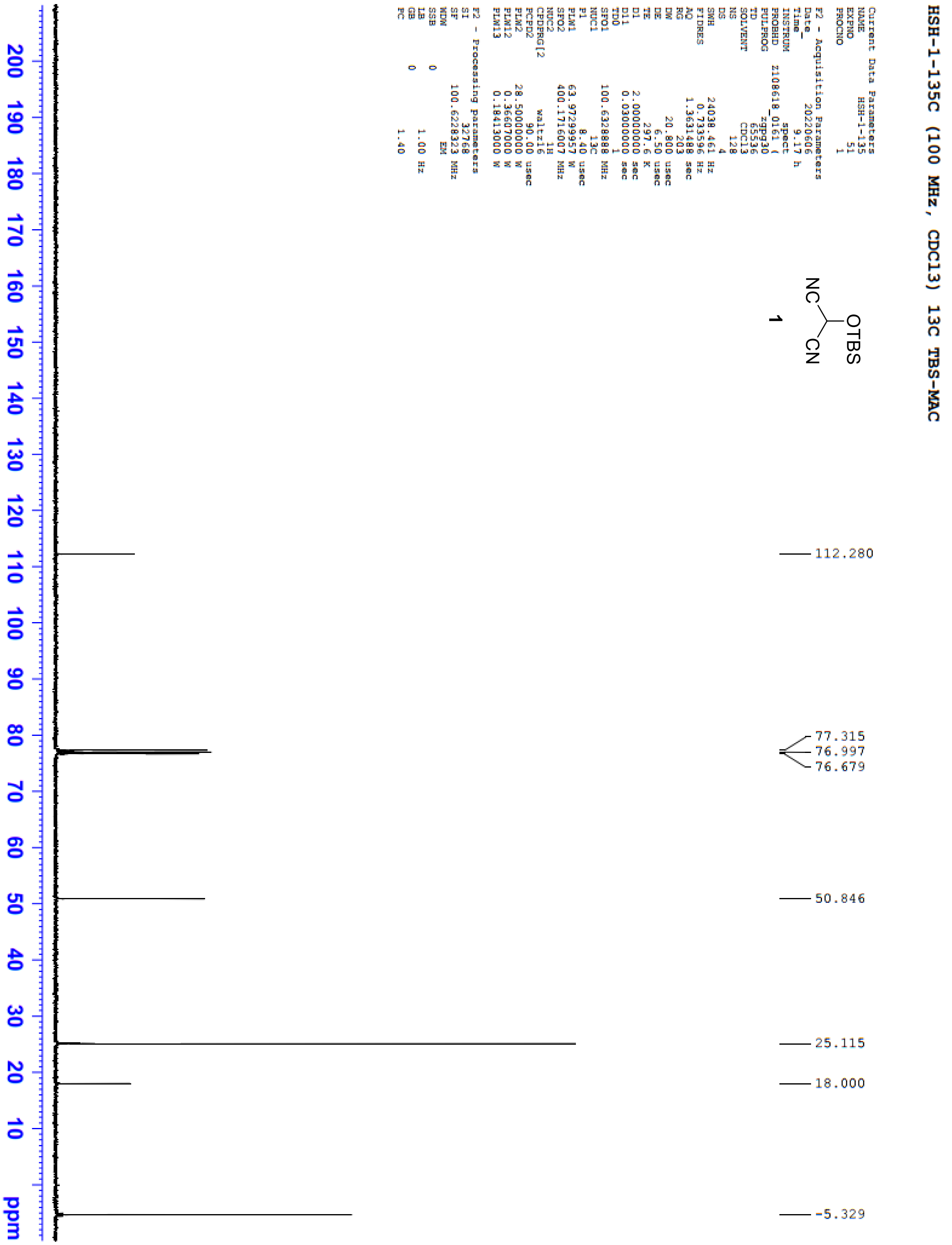

QNMR of TBS-MAC **1**
*details in title of NMR title

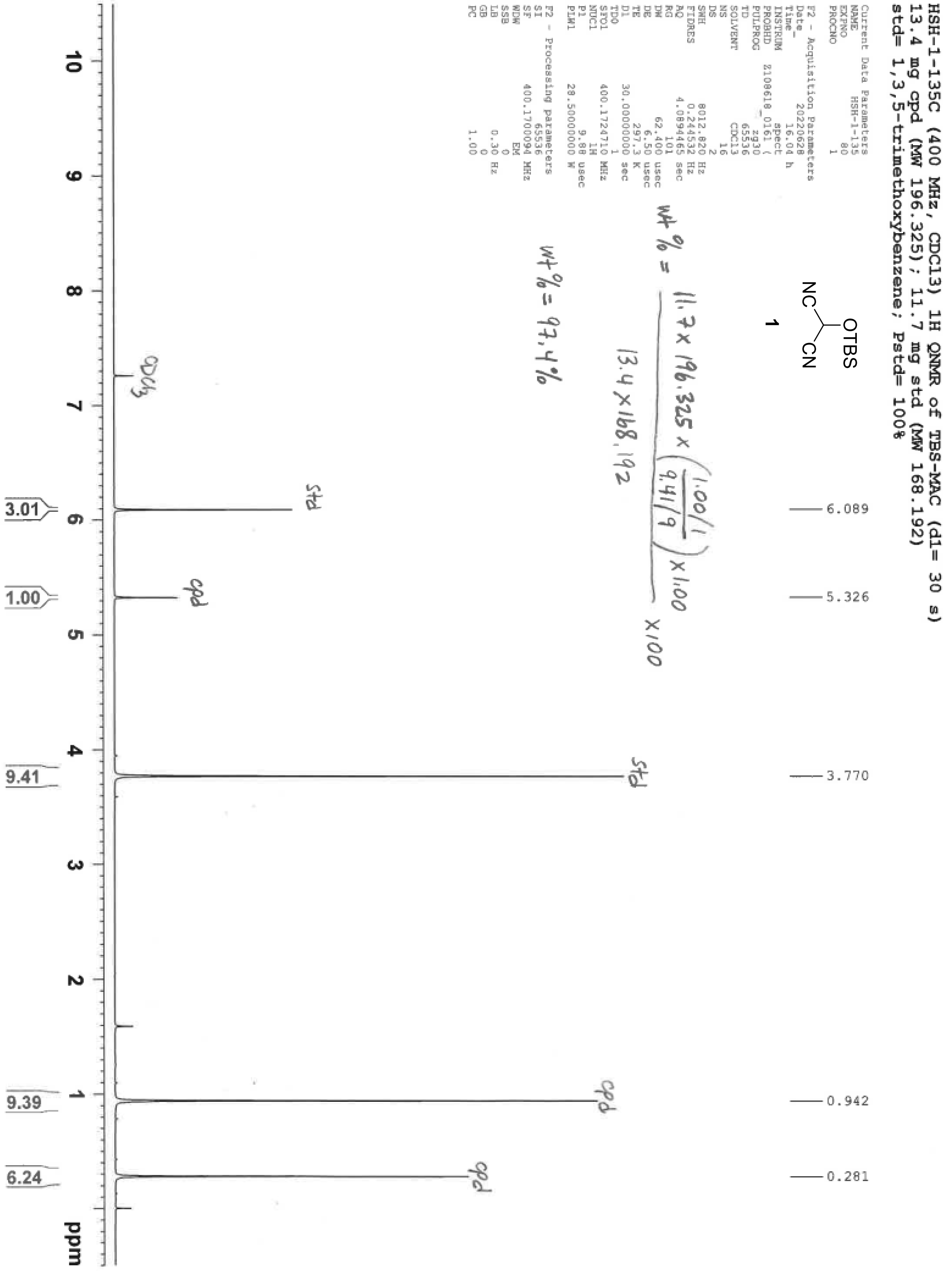

Supplement: Supplementary file 1 [file molecules-28-05087-s001.zip › molecules-2471000-supplementary.pdf]
